# Efficacy and safety of the integration of traditional Chinese medicine and western medicine in the treatment of diabetes-associated cognitive decline: a systematic review and meta-analysis

**DOI:** 10.3389/fphar.2023.1280736

**Published:** 2023-11-22

**Authors:** Jianan Su, Guiyan Sun, Jiren An, Yuhan Ao, Jing Li, Zihan Shen, Lanyi Zhang, Shiheng Zhang, Yufeng Yang, Yan Shi

**Affiliations:** ^1^ Liaoning Key Laboratory of Chinese Medicine Combining Disease and Syndrome of Diabetes, Liaoning University of Traditional Chinese Medicine, Shenyang, China; ^2^ College of Integrative Chinese and Western Medicine, Hebei University of Chinese Medicine, Shijiazhuang, China; ^3^ College of Traditional Chinese Medicine, Liaoning University of Traditional Chinese Medicine, Shenyang, China; ^4^ College of First College, Liaoning University of Traditional Chinese Medicine, Shenyang, China

**Keywords:** diabetes-associated cognitive decline, traditional Chinese medicine, meta-analysis, systematic review, grade evaluation

## Abstract

**Objective:** In order to offer possible therapeutic treatment evidence for diabetes-associated cognitive decline (DACD), we thoroughly evaluated the effectiveness and safety of combining Traditional Chinese Medicine (TCM) and Western Medicine (WM) in the current study.

**Methods:** The present study employed a comprehensive search strategy across multiple databases, namely, PubMed, EMBASE, Web of Science, the Cochrane Library, China National Knowledge Infrastructure (CNKI), Wanfang Data, Chinese Scientific Journals Database (VIP), and Chinese Biomedical Literature Database (CBM), to identify relevant articles published until July 2023. Subsequently, a systematic review and meta-analysis of randomized controlled trials (RCTs) were conducted to assess the efficacy and safety of integrating TCM with WM for the treatment of DACD. The literature included in this study was assessed using the GRADE criteria and the Cochrane Handbook for Systematic Reviews of Interventions. Statistical analysis was conducted using RevMan 5.4 software.

**Results:** A total of 20 RCTs involving 1,570 patients were ultimately included in this meta-analysis. The pooled results demonstrated that the integration of TCM and WM therapy significantly enhanced the overall effectiveness rate compared to WM therapy alone [OR = 4.94, 95% CI (3.56, 6.85), *p* < 0.00001]. Additionally, the combination therapy resulted in reductions in fasting blood glucose [MD = −0.30, 95% CI (−0.49, −0.10), *p* = 0.003], HbA1c [MD = −0.71, 95%CI (−1.03, −0.40), *p* < 0.00001], TNF-α levels [MD = −8.28, 95%CI (−13.12, −3.44), *p* = 0.0008], and TCM Syndrome Score [MD = −5.97, 95%CI (−9.06, −2.88), *p* = 0.0002]. Meanwhile, the combination therapy had a positive effect on MoCA Score [MD = 2.52, 95% CI (1.75, 3.30), *p* < 0.00001], and MMSE Score [MD = 2.31, 95% CI (1.33, 3.29), *p* < 0.00001]. In addition, the safety of the combination therapy was comparable to that of the WM alone [OR = 0.40, 95% CI (0.12, 1.31), *p* = 0.13].

**Conclusion:** The integration of TCM and WM therapy outperformed WM alone in DACD treatment. Simultaneously, the combination therapy could improve the therapeutic effect on blood glucose, cognitive function, and inflammation to a certain extent with few adverse effects. However, given the constraints imposed by the quality limitations of the incorporated studies, as well as the potential presence of reporting bias, it is imperative that our findings be substantiated through rigorous, large-scale, randomized controlled trials of superior quality in the future.

## Graphical Abstract

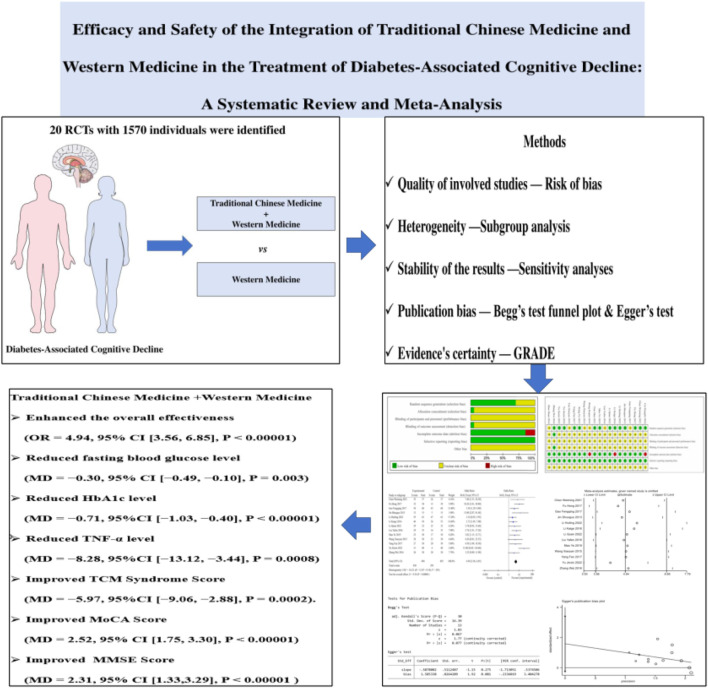

## 1 Introduction

Diabetes mellitus (DM) is a metabolic disorder that is becoming more prevalent, marked by chronic hyperglycemia and a deficiency in insulin production or sensitivity (Banday et al., 2020). Diabetes mellitus is spreading globally, with an estimated 366 million people in the world by 2030, and thus it has become an urgent and worldwide public health issue threatening human health (Aschner et al., 2021). Neurodegenerative disease is increasingly recognized as an additional complication of diabetes mellitus, in addition to the well-established microvascular and macrovascular complications (Mauricio et al., 2020). Extensive research has demonstrated a correlation between type 2 diabetes mellitus (T2DM) and the development of neurodegenerative diseases such as Alzheimer’s disease, vascular dementia, and cognitive impairment (Khaledi et al., 2019; Sutherland et al., 2017; Stoeckel et al., 2016). Notably, T2DM is closely linked to a heightened risk of cognitive impairment, with approximately 60%–70% of T2DM patients experiencing cognitive dysfunction (Gupta et al., 2022; Chatterjee et al., 2016).

Diabetes-associated cognitive decline (DACD) is a prevalent neurological complication of T2DM that primarily presents as cognitive deficits, involving attention and executive functions (Chen et al., 2018). Furthermore, it has gradually become a worldwide health concern due to the advancement of living standards, the acceleration of aging, and the changes in lifestyle (Srikanth et al., 2020). Unfortunately, we remain a shallow understanding on the exact pathogenesis of DACD until now, and thus the range of treatment options is constrained when there is a lack of efficacious and targeted therapeutic medications (Chinese Society of Endocrinology, 2022). The current approach to treating DACD primarily emphasizes the comprehensive management of multiple risk factors, such as blood glucose control, enhancement of cerebral blood supply, and preservation of cognitive function. However, the intricacy of multidrug regimens may engender the likelihood of nonadherence among patients, whereas the prolonged utilization of hypoglycemic agents may escalate the occurrence of adverse effects, including gastrointestinal discomfort, weight gain, and hepatic dysfunction (Chinese Society of Endocrinology, 2021). In light of the unsatisfactory outcomes associated with current treatments, clinicians are taking a closer look at Traditional Chinese Medicine (TCM) as an adjuvant or alternative treatment.

Traditional Chinese medicine therapy is extensive and profound, which has been inherited and applied for more than 2,000 years, is the treasure of Chinese culture. The theory of TCM promotes the principle of “harmony between man and nature” and strives for comprehensive treatment. The people-centered, holistic, and multitarget strategies employed by TCM offer distinct benefits in managing intricate conditions, including DM (Marín-Peñalver et al., 2016). There is no definite disease name of DACD in TCM literature, but according to its clinical characteristics and performance, it is classified as “Xiao Ke” combined with “Chi Dai” or “Jian Wang.” In recent years, a substantial body of preclinical (*in vivo*/*in vitro*) experiments and clinical observation studies has provided evidence supporting the therapeutic efficacy of the integration of traditional Chinese medicine and western medicine in the treatment of DACD. Meanwhile, the potential therapeutic mechanism is still being improved and supplemented, including oxidative stress (Hao et al., 2019), gut microbiota (Zheng et al., 2022), autophagy (Tian et al., 2022), neuroinflammation (Shi et al., 2021), etc. However, most of the published clinical studies are small single-center clinical trials, lacking high-quality systematic evaluation and review of clinical treatment. Efficacy and safety of this treatment must therefore be demonstrated through evidence-based studies. Given the aforementioned constraints, we conducted an extensive review of both domestic and foreign literature to impartially assess the clinical effectiveness and safety of combining TCM and Western Medicine (WM) for patients with DACD, aiming to shed light on clinical treatment approaches.

## 2 Materials and methods

The review procedure was carried out in accordance with PRISMA guidelines, and it has been submitted to the International Platform of Registered Systematic Review and Meta-analysis Protocols (INPLASY) under registration number INPLASY202320072.

### 2.1 Literature search strategy

To identify relevant studies on biological therapeutic interventions for DACD, an extensive search was conducted across several databases, including China National Knowledge Infrastructure (CNKI), Wanfang Database, Chinese Scientific Journals Database (VIP), Chinese Biomedical Literature Database (CBM), PubMed, EMBASE, Web of Science, and Cochrane Library. The retrieval period encompassed the entire duration of databases up until July 2023. No restrictions were placed on language, systemic conditions of participants, or publication year within the scope of this study. The search strategy employed a comprehensive approach, utilizing both MeSH terms and keywords, with a specific emphasis on the topics of “diabetic cognitive impairment” and “traditional Chinese medicine and western medicine.” Additionally, the search encompassed intervention measures and diseases associated with these topics, such as proprietary Chinese medicine, Chinese medicine herbs, herbal medicine, diabetic cognitive dysfunction, and diabetic encephalopathy etc. Furthermore, a manual search was conducted in the journal literature available at the Liaoning University of Traditional Chinese Medicine library to complement the initial search and identify any potential omissions. The specific search strategy of each database was shown in Supplementary Material S1.

### 2.2 Inclusion criteria

The inclusion criteria were established using the PICOS framework, encompassing participant, intervention, comparison, outcomes, and study design.

#### 2.2.1 Types of participants

The study did not impose any limitations based on age, gender, or race. The participants included individuals who had received a diagnosis of DACD based on a well-defined definition or internationally recognized diagnostic criteria.

#### 2.2.2 Types of interventions

The intervention implemented in this study entailed the integration of TCM and WM. In the treatment group, TCM treatment was exclusively employed as the positive intervention, contrasting with the control group. No limitations were imposed on the dosage or duration of medication.

#### 2.2.3 Types of comparison

The use of WM treatment, including hypoglycemic agents, insulin, nimodipine, donepezil, and others, has been shown to effectively lower blood glucose levels and enhance cognitive function. The control groups in the studies employed the same specifications and dosage of WM as the treatment groups.

#### 2.2.4 Types of outcomes

The primary outcome measure was the total effective rate, while secondary outcomes included fasting plasma glucose (FPG), glycated hemoglobin (HbA1c), MoCA score, MMSE score, TNF-α, TCM syndrome score, and adverse reactions. All included literature reported at least two results from the aforementioned outcomes.

#### 2.2.5 Types of study design

All randomized controlled trials (RCTs) that reported the utilization of TCM in combination with WM for the treatment of DACD were included in this study. No restrictions were placed on publication status or language.

### 2.3 Exclusion criteria

The exclusion criteria were set as followed. 1) Non-RCTs or animal studies. 2) Control group included methods of TCM, such as acupuncture, Chinese patent medicine, herbal extracts and so on. 3) Repeated publication or repeated clinical data. 4) Original and unpublished data that could not be obtained and extracted after contacting the authors. 5) Outcome effect was not clear: The data were incomplete, the outcome effect was not clear, the statistical method was incorrect, and the data could not provide the mean and standard deviation.

### 2.4 Baseline characteristics and assessment of included studies

The data extraction process for the studies was conducted by two independent reviewers (Jiren An and Guiyan Sun). To facilitate this process, a study-specific spreadsheet was created in Excel, encompassing variables such as authors, publication date, country, study design, sample size, average age, gender, intervention measures, follow-up duration, and outcome measures. Subsequently, all data were cross-verified and imported into Rev Man software (V.5.4). The Cochrane Handbook for Systematic Reviews was utilized to evaluate the risk of bias in all studies included in this analysis. These studies were categorized as having a low, high, or unclear risk of bias based on seven specific criteria: 1) random sequence generation; 2) allocation concealment; 3) blinding of participants and personnel; 4) blinding of outcome assessors; 5) incomplete outcome data; 6) selective reporting; and 7) other potential risks of bias. In cases where there was disagreement, a third reviewer investigator (Yufeng Yang) was consulted to reach a resolution.

### 2.5 Data analysis and synthesis

Statistical analyses were performed using the Review Manager program (version 5.4.1, The Cochrane Collaboration, The Nordic Cochrane Centre, Copenhagen, Denmark) and Stata software (version 16, The Stata Corporation, College Station, Texas, United States). To measure the effect size, Risk ratio (RR) with 95% confidence intervals (CIs) was used as an evaluation index for dichotomous data, including total effective rate and adverse reaction. Mean difference (MD) or standardized mean difference (SMD) with 95% CI was used as an evaluation index for continuous data, including FPG, HbA1c, MoCA score, MMSE score, TNF-α, AND TCM syndrome score.

Heterogeneity among the outcomes of the included studies was analyzed using the Cochrane Q test, while the magnitude of heterogeneity was determined quantitatively in combination with I^2^ (Higgins and Thompson, 2002). If there was no heterogeneity (*p* > 0.05, I^2^ ≤ 50%), the fixed-effect model was selected; if there was heterogeneity (*p* ≤ 0.05, I^2^ > 50%), the random-effect model was used. *p* < 0.05 was considered statistically significant.

### 2.6 Subgroup analyses, sensitivity analyses and publication bias

Subgroup analysis was performed on the clinical characteristics to investigate the causes of clinical heterogeneity. By excluding one study at a time, sensitivity analysis was used to examine whether low-quality studies affected the robustness and stability of the overall meta-analysis. Begg’s test funnel plot and Egger’s test were used to evaluate publication bias.

### 2.7 Evaluation of the certainty of the evidence

The Grading of Recommendations Assessment, Development, and Evaluation (GRADE) tool was used to evaluate the quality of cumulative evidence in this review (Goldet and Howick, 2013). By assessing the factors such as the risk of bias, inconsistency, and indirectness, the certainty level of evidence was judged using four categories of “very low,” “low,” “moderate,” and “high.”

## 3 Results

### 3.1 Literature retrieval process and results

**FIGURE 1:**
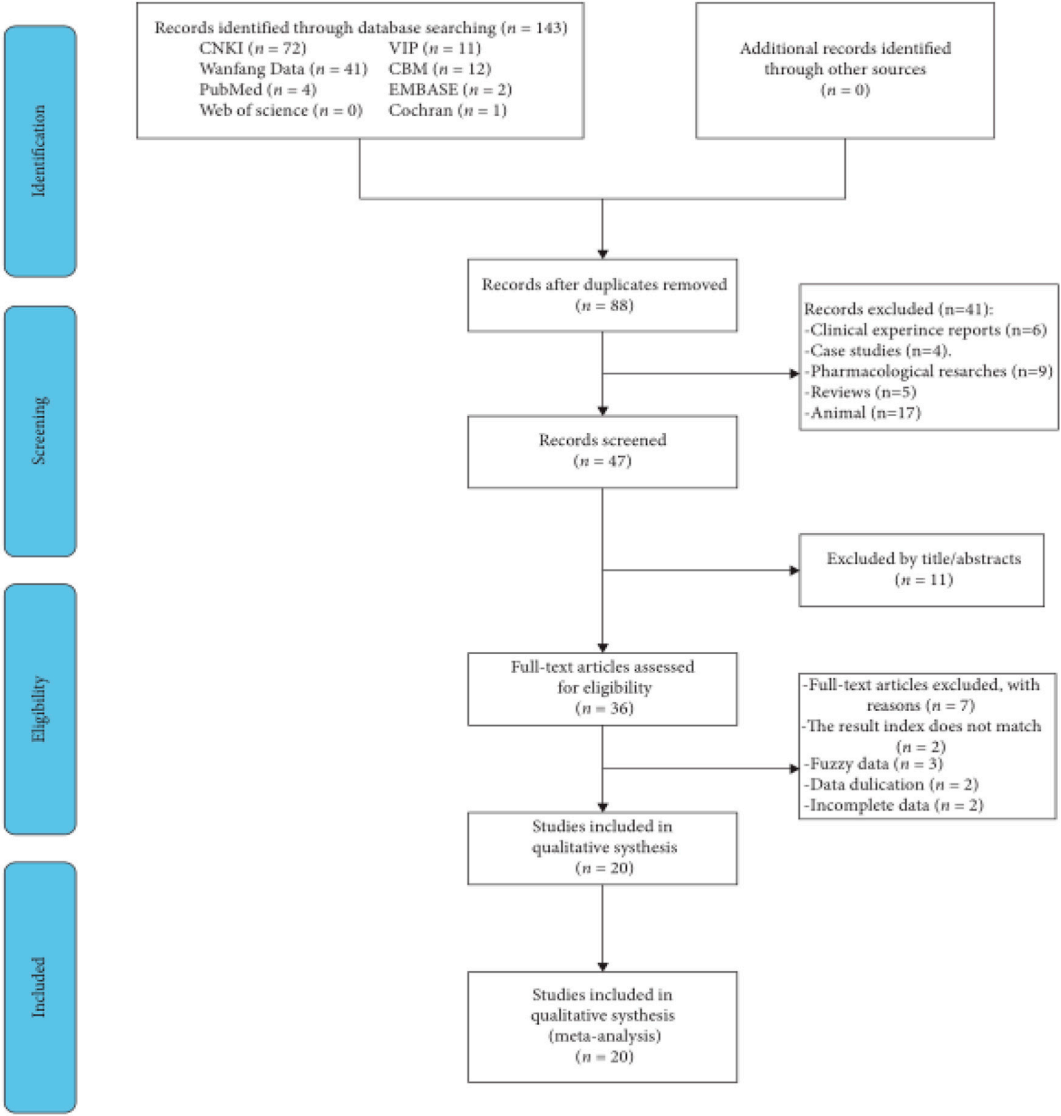
Flow chart for study selection process.

A preliminary literature search turned up a total of 143 publications, and 20 articles (Cai, 2016; Chen et al., 2021; Fu et al., 2017; Gao et al., 2017; Jin et al., 2015; Li, 2022; Li, 2016; Li et al., 2022; Liu et al., 2016; Mao et al., 2019; Tian et al., 2021; Wang et al., 2016; Wang, 2015; Wang et al., 2015; Yan and Guan, 2019; Yang, 2017; Yu, 2018; Yu et al., 2022; Zhang, 2016; Zhao et al., 2014) that qualified were subsequently found. Figure 1 displayed the search procedures.

### 3.2 Study characteristics and assessment of risk of bias

All studies were published before July 2023 and conducted in China. Totally, this meta-analysis involved 1,570 participants, of which 788 participants were designated to the experimental group while 782 patients were assigned to the control group. The sample size varied from 30 to 126, with a mean patient age ranging from 59 to 80 years old. and the average course of disease ranged from less than 12 months to more than 17 years. Regarding of the treatment measures, the control group adopted the therapy of western medicine alone, whereas the experimental group received combined treatment with TCM and WM. Specially, western medicine included hypoglycemic drug, antihypertensive drug, hypolipidemic drug, donepezil, nimodipine, oxiracetam citicoline sodium tablets, aspirin enteric-coated tablet, and insulin etc. Meanwhile, a total of 17 types of Chinese herbal medicine were used in the 20 RCTs, all of which were multi-herbal medicines, including Bushen Huoxue decoction, Bushen Huoxue Kaiqiao recipe, Bushen Huoxue recipe, Bushen Jiannao granules, Bushen Jianpi Huoxue recipe, Bushen Yiqi Huoxue recipe, Jiaotai pill, Naoling decoction, Rehmannia decoction, Shenqi Yizhi Jiannao recipe, Xuefu Zhuyu decoction, Yangyi Yizhi decoction, Yiqi Bushen Huoxue recipe, Yiqi Yangyin Huoxue recipe, Yishen Huoxue recipe, Yizhi Mixture, Zishen Qushi Huatan recipe. Among the 20 included studies, the duration of treatment ranged from 2 months to 12 months. In terms of the outcomes, 13 studies mentioned the total effective rate, 10 studies reported the FPG level, 11 studies reported the HbA1c level, 12 studies reported the MoCA score, 11 studies reported the MMSE score, 3 studies reported the TNF-α level, 7 studies reported the TCM syndrome score, and 12 studies reported the adverse reaction. The experimental group and the control group had equivalent pre-treatment data (such as age, sex ratio, outcome indicators, etc.). The basic characteristics of the included studies were summarized in Table 1, and components of Chinese herbal medicine used in the included studies were presented in Table 2.

**TABLE 1:** Summary of included studies.

Author (year)	Sample size E/C	Gender (M/F)		Course of disease	Mean age (y) E/C	Co- intervention	Intervention		Duration (months)	Outcomes
		E	C	E/C			E	C		
Cai 2016	24/24	15/9	14/10	9.0 ± .8/9.2 ± 2.0	66.2 ± 4.2/66.8 ± 4.5	NR	Yishen Huoxue Recipe + C	Conventional treatment (oral hypoglycemic drug or insulin) and brain neuroprotective drug	3	⑤⑦
Chen et al., 2021	37/37	20/17	19/18	NR	71.56 ± 7.13/71.18 ± 7.12	Lifestyle intervention	Naoling Decoction + C	Conventional treatment (oral hypoglycemic drug or insulin) and donepezil (5 mg, qd)	6	①②③④⑤
Fu et al., 2017	40/40	20/20	19/21	17.7 ± 8.4/15.5 ± 7.8	67.2 ± 8.5/67.4 ± 9.8	NR	Bushen Huoxue Recipe + C	Conventional treatment (oral hypoglycemic drug or insulin + antihypertensive drug + hypolipidemic drug) and nimodipine (30 mg, tid)	3	①②③④⑧
Gao et al., 2017	60/60	36/24	34/26	NR	62.40 ± 5.95/62.08 ± 5.92	NR	Zishen Qushi Huatan Recipe + C	Donepezil (5 mg, qd)	3	①④⑤⑧
Jin et al., 2015	15/15	7/8	9/6	NR	62.5 ± 5.4/63.0 ± 5.6	NR	Bushen Huoxue Kaiqiao Recipe + C	Nimodipine (30 mg, tid)	3	①④⑦⑧
Li et al., 2022	63/63	32/31	35/28	7.74 ± 3.82/7.31 ± 3.28	62.73 ± 5.12/61.46 ± 6.17	NR	Yiqi Yangyin Huoxue Recipe + C	Conventional treatment (oral hypoglycemic drug or insulin + antihypertensive drug) and donepezil (5 mg, qd)	6	①②③④⑤⑥
Li 2016	54/53	30/24	29/24	7.5 ± 2.3/7.4 ± 2.3	61.3 ± 4.7/61.2 ± 4.7	NR	Bushen Huoxue Kaiqiao Recipe + C	Nimodipine (10 mg, tid)	3	①④
Li 2022	32/32	21/11	22/10	5.34 ± 0.88/5.70 ± 0.97	59.03 ± 7.15/60.06 ± 6.58	Lifestyle intervention	Rehmannia Decoction + C	Conventional treatment (oral hypoglycemic drug) and donepezil (5 mg, qd)	6	①②③④⑤⑦⑧
Liu et al., 2016	35/35	16/19	18/17	6.5 ± 2.7/7.1 ± 2.9	65.6 ± 7.6/68.2 ± 6.9	NR	Yiqi Bushen Huoxue Recipe + C	Conventional treatment (oral hypoglycemic drug or insulin + antihypertensive drug)	3	①⑤
Mao et al., 2019	30/30	19/11	21/9	10.56 ± 1.43/9.21 ± 1.65	60.32 ± 4.51/63.25 ± 4.03	Lifestyle intervention	Yangyi Yizhi Decoction + C	Conventional treatment (oral hypoglycemic drug or insulin + antihypertensive drug) and nimodipine (30 mg, tid)	2	①⑤
Tian et al., 2021	40/40	23/17	22/18	8.2 ± 2.8/8.5 ± 3.2	69.5 ± 5.3/69.2 ± 5.4	NR	Jiaotai Pill + C	Conventional treatment (oral hypoglycemic drug or insulin) + oxiracetam (0.8 g, tid)	6	④⑥
Wang et al., 2016	53/52	34/19	37/15	7.4 ± 2.4/7.5 ± 2.5	60.2 ± 10.1/61.6 ± 10.5	Lifestyle intervention	Bushen Yiqi Huoxue Recipe + C	Metformin (0.5 g, tid) or insulin + citicoline sodium tablets (0.2 g, tid)	12	②③
Wang 2015	30/30	17/13	16/14	11.1 ± 2.9/11.3 ± 2.8	69.3 ± 3.5/69.5 ± 3.4	Lifestyle intervention	Bushen Jiannao Granules + C	Conventional treatment (oral hypoglycemic drug or insulin + antihypertensive drug + hypolipidemic drug) and nimodipine (30 mg, tid)	2	①⑤
Wang et al., 2015	57/57	29/28	31/26	7.7 ± 4.4/7.5 ± 4.8	61.1 ± 7.8/60.4 ± 7.5	NR	Bushen Huoxue Decoction + C	Conventional treatment (oral hypoglycemic drug or insulin + antihypertensive drug + hypolipidemic drug), nimodipine (30 mg, tid) and bayaspirin enteric-coated (100 mg, qd)	2	②③④⑥
Yan and Guan 2019	30/30	18/12	16/14	11.56 ± 4.39/9.83 ± 5.79 (months)	71.37 ± 4.66/70.56 ± 5.26	NR	Yizhi Mixture + C	Conventional treatment (oral hypoglycemic drug or insulin) and citicoline sodium tablets (0.2 g, tid)	2	②③⑤
Yang 2017	30/30	16/14	14/16	6.83 ± 1.64/7.33 ± 2.04	61.63 ± 6.10/62.17 ± 5.27	Lifestyle intervention	Shenqi Yizhi Jiannao Recipe + C	Metformin (500 mg, qd), shagliptin (5 mg, qd) and nimodipine (30 mg, tid)	3	①②③④⑤⑦
Yu 2018	26/25	18/8	16/9	NR	67/66.5	NR	Xuefu Zhuyu Decoction + C	Conventional treatment (oral hypoglycemic drug or insulin)	3	②③
Yu et al., 2022	40/40	23/19	22/20	11.69 ± 2.84/11.05 ± 3.93	71.67 ± 6.09/71.10 ± 6.12	Lifestyle intervention	Bushen Jianpi Huoxue Recipe + C	Conventional treatment (antihypertensive drug + hypolipidemic drug), metformin (500 mg, qd), gliclazide (80 mg, bid, if necessary) and aspirin enteric-coated tablet (100 mg, qd)	3	①③④⑦⑧
Zhang 2016	32/33	17/13	14/16	NR	79.67 ± 7.68/80.62 ± 6.42	Lifestyle intervention	Yizhi Mixture + C	Conventional treatment (oral hypoglycemic drug or insulin) and oxiracetam injection (4.0 g, qd)	3	①②③⑤⑦
Zhao et al., 2014	62/58	44/18	40/18	NR	65.5 ± 5.9/64.0 ± 5.8	NR	Bushen Huoxue Kaiqiao Recipe + C	Aspirin (100 mg, qd)	6	④⑦⑧

E, Experimental group; C, Control group; NR, Not reported. Outcome: ① Total effective rate; ② FPG; ③ HbAlc; ④ MoCA, score; ⑤ MMSE, score; ⑥ TNF-α; ⑦ TCM, syndrome score; ⑧ Adverse reaction.

**TABLE 2:** Components of Chinese herbal medicine used in the included studies.

Study	Prescription name	Source	Extraction process	Compositions	Usage of preparations	Preparations	Quality control reported?	Chemical analysis reported?
Cai 2016	Yishen Huoxue Recipe	NR	NR	Hornes of Cervus nippon Temminck [Cervidae; Cervi Cornus Colla] 20g, Rehmannig glutinosa Libosch. [Orobanchaceae; Rehmanniae radix praeparata] 20g, Reynoutria multiflora (Thunb.) Moldenke [Polygonaceae; Polygoni multiflori radix] 20g, Lycium barbarum L. [Solanaceae; Lycii fructus] 15g, Polygala tenuifolia Willd. [Polygalaceae; Polygalae Radix] 10 g, Carthamus tinctorius L. [Asteraceae; Carthami Flos] 10 g, Ziziphus jujuba Mill.var.spinosa (Bunge) Hu ex H.F.Chou [Rhamnaceae; Ziziphi Spinosae Semen] 20 g, Ligusticum chuanxiong Hort. [Apiaceae; Chuanxiong rhizoma] 12 g, Salvia miltiorrhiza Bunge [Lamiaceae; Salviae Miltiorrhizae Radix Et Rhizoma] 20g, Acorus calamus var. angustatus Besser [Acoraceae; Acori tatarinowii rhizoma] 12g, Alpinia oxyphylla Miq. [Zingiberaceae; Alpiniae Oxyphyllae Fructus] 12 g	1 package bid po	Decoction	NR	NR
Chen et al., 2021	Naoling Decoction	Shaanxi Traditional Chinese Medicine Hospital	Partially reported^c^	Epimedium brevicornu Maxim. [Ginkgoaceae; Epimedii Folium] 10 g, Rhodiola rosea L. [Crassulaceae; Rhodiolae Crenulatae Radixet Rhizoma] 15g, Cnidium monnieri (L.) Cuss. [Apiaceae; Cnidii Fructus] 10 g, Reynoutria multiflora (Thunb.) Moldenke [Polygonaceae; Polygoni multiflori radix] 20g, Cullen corylifolium (L.) Medik. [Fabaceae; Psoraleae Fructus] 15g, Panax ginseng C.A.Mey. [Araliaceae; Ginseng radix et rhizoma] 15g, Eucommia ulmoides Oliv. [Eucommiaceae; Eucommiae Cortex] 15g, Acorus calamus var. angustatus Besser [Acoraceae; Acori tatarinowii rhizoma] 15 g	200 ml bid po	Decoction	NR	NR
Fu et al., 2017	Bushen Huoxue Recipe	Peking University First Hospital	Partially reported^c^	Cuscuta chinensis Lam. [Convolvulaceae; Cuscutae Semen] 15 g, Lycium barbarum L. [Solanaceae; Lycii fructus] 15 g, Rubus chingii Hu [Rosaceae; Rubi Fructus] 10 g, Schisandra chinensis (Turcz.) Baill. [Schisandraceae; Schisandrae chinensis fructus] 6 g, Plantago asiatica L. [Plantaginaceae; Plantaginis Herba] 5g, Epimedium brevicornu Maxim. [Ginkgoaceae; Epimedii Folium] 10 g, Hirudo niponica Whitman [Hirudo; Hirudo] 3 g	1 package bid po	Decoction	NR	NR
Gao et al., 2017	Zishen Qushi Huatan Recipe	NR	NR	Panax ginseng C.A.Mey. [Araliaceae; Ginseng radix et rhizoma] 20 g, Dioscorea oppositifolia L. [Dioscoreaceae; Dioscoreae rhizoma] 15g, Poria cocos (Schw.) Wolf [Polyporaceae; Poria] 15 g, Salvia miltiorrhiza Bunge [Lamiaceae; Salviae Miltiorrhizae Radix Et Rhizoma] 15 g, Cistanche deserticola Ma [Orobanchaceae; Cistanches Herba] 15 g, Pinellia ternata (Thunb.) Makino [Araceae; Pinelliae rhizoma] 10 g, Amomum longiligulare T.L.Wu [Zingiberaceae; Amomi Fructus] 10 g, Acorus calamus var. angustatus Besser [Acoraceae; Acori tatarinowii rhizoma] 8 g, Glycyrrhiza uralensis Fisch. ex DC. [Fabaceae; Glycyrrhizae radix et rhizoma] 8 g	1 package bid po	Decoction	NR	NR
Jin et al., 2015	Bushen Huoxue Kaiqiao Recipe	NR	NR	Cistanche deserticola Ma [Orobanchaceae; Cistanches Herba] 10g, Acorus calamus var. angustatus Besser [Acoraceae; Acori tatarinowii rhizoma] 5g, Panax notoginseng (Burkill) F.H.Chen [Araliaceae; Notoginseng radix et rhizoma] 2.5 g	1 package tid po	Decocted preparation	NR	NR
Li 2022	Yiqi Yangyin Huoxue Recipe	NR	NR	Panax quinquefolium L. [Araliaceae; Panacis Quinquefolii Radix] 10 g, Rehmannig glutinosa Libosch. [Orobanchaceae; Rehmanniae radix praeparata] 15 g, Ophiopogon japonicus (Thunb.) Ker Gawl. [Asparagaceae; Ophiopogonis radix] 10 g, Schisandra chinensis (Turcz.) Baill. [Schisandraceae; Schisandrae chinensis fructus] 6 g, Carthamus tinctorius L. [Asteraceae; Carthami Flos] 8 g, Ligusticum chuanxiong Hort. [Apiaceae; Chuanxiong rhizoma] 8 g, Atractylodes macrocephala Koidz. [Asteraceae; Atractylodis macrocephalae rhizoma] 15 g, Polygala tenuifolia Willd. [Polygalaceae; Polygalae Radix] 10 g, Actaea cimicifuga L. [Ranunculaceae; Cimicifugae rhizoma] 6 g, Glycyrrhiza uralensis Fisch. ex DC. [Fabaceae; Glycyrrhizae radix et rhizoma] 5 g	1 package bid po	Decoction	NR	NR
Li 2016	Bushen Huoxue Kaiqiao Recipe	NR	Partially reported^c^	Polygonatum sibiricum Redouté [Asparagaceae; Polygonati Rhizoma] 30g, Rehmannig glutinosa Libosch. [Orobanchaceae; Rehmanniae radix praeparata] 15g, Rehmannia glutinosa (Gaertn.) DC. [Orobanchaceae; Rehmanniae radix praeparata] 15 g, Cistanche deserticola Ma [Orobanchaceae; Cistanches Herba] 15 g, Polygala tenuifolia Willd. [Polygalaceae; Polygalae Radix] 15 g, Acorus calamus var. angustatus Besser [Acoraceae; Acori tatarinowii rhizoma] 5 g, Ginkgo biloba L. [Zingiberaceae; Ginkgo Folium] 10 g, Actaea cimicifuga L. [Ranunculaceae; Cimicifugae rhizoma] 10 g, Panax notoginseng (Burkill) F.H.Chen [Araliaceae; Notoginseng radix et rhizoma] 2.5 g, Glycyrrhiza uralensis Fisch. ex DC. [Fabaceae; Glycyrrhizae radix et rhizoma] 6 g	1 package tid po	Decoction	NR	NR
Li et al., 2022	Rehmannia Decoction	The First Affiliated Hospital of Heilongjiang University of Traditional Chinese Medicine	Partially reported^c^	Rehmannia glutinosa (Gaertn.) DC. [Orobanchaceae; Rehmanniae radix praeparata]15g, Gynochthodes officinali How [Rubiaceae; Morindae Officinalis Radix] 15g, Cornus officinalis Siebold & Zucc. [Cornaceae; Corni Fructus] 15g, Dendrobium nobile Lindl. [Orchidaceae; Dendrobii Caulis] 15g, Cistanche deserticola Ma [Orobanchaceae; Cistanches Herba] 15g, Aconitum carmichaelii Debx [Ranunculaceae; Aconiti Lateralis Radix Praeparata] 15g, Polygala tenuifolia Willd. [Polygalaceae; Polygalae Radix] 15g, Neolitsea cassia (L.) Kosterm. [Lauraceae; Cinnamomi Cortex] 15g, Schisandra chinensis (Turcz.) Baill. [Schisandraceae; Schisandrae chinensis fructus] 15g, Poria cocos (Schw.) Wolf [Polyporaceae; Poria] 15g, Ophiopogon japonicus (Thunb.) Ker Gawl. [Asparagaceae; Ophiopogonis radix] 15 g, Acorus calamus var. angustatus Besser [Acoraceae; Acori tatarinowii rhizoma] 15 g, Mentha canadensis L. [Lamiaceae; Menthae Haplocalycis Herba] 10 g, Zingiber offcinale Rosc. [Zingiberaceae; Zingiberis Rhizoma Recens] 5 g, Ziziphus jujuba Mill. [Rhamnaceae; Jujubae Fructus] 5 g	1 package bid po	Decoction	NR	NR
Liu et al., 2016	Yiqi Bushen Huoxue Recipe	First Clinical Hospital Affiliated to Jilin Province Academy of Traditional Chinese Medicine	Partially reported^c^	Panax ginseng C.A.Mey. [Araliaceae; Ginseng radix et rhizoma], Cervus nippon Temminck [Cervidae; Cervi Cornu Pantotrichum], Rehmannig glutinosa Libosch. [Orobanchaceae; Rehmanniae radix praeparata], Panax notoginseng (Burkill) F.H.Chen [Araliaceae; Notoginseng radix et rhizoma], Paeonia suffruticosa Andr. [Paeoniaceae; Moutan Cortex], Acorus calamus var. angustatus Besser [Acoraceae; Acori tatarinowii rhizoma], Polygala tenuifolia Willd. [Polygalaceae; Polygalae Radix]	NR	Granule	NR	NR
Mao et al., 2019	Yangyi Yizhi Decoction	Affiliated Hospital of Hunan Academy of Traditional Chinese Medicine	Partially reported^c^	Astragalus mongholicus Bunge [Fabaceae; Astragali radix] 30g, Trichosanthes kirilowii Maxim. [Cucurbitaceae; Radix Trichosanthis] 30g, Dioscorea oppositifolia L. [Dioscoreaceae; Dioscoreae rhizoma] 15g, Rehmannig glutinosa Libosch. [Orobanchaceae; Rehmanniae radix praeparata] 15g, Cornus officinalis Siebold & Zucc. [Cornaceae; Corni Fructus] 15g, Salvia miltiorrhiza Bunge [Lamiaceae; Salviae Miltiorrhizae Radix Et Rhizoma] 15g, Cistanche deserticola Ma [Orobanchaceae; Cistanches Herba] 15 g, Trigonella foenum-graecum L. [Fabaceae; Semen Trigonellae] 15 g, Ophiopogon japonicus (Thunb.) Ker Gawl. [Asparagaceae; Ophiopogonis radix] 12 g, Euonymus alatus (Thunb.) Siebold [Celastraceae; Ramulus euonymi] 10 g, Pheretima aspergillum (E. Perrier) [Megascolecidae; Pheretima] 10 g, Polygala tenuifolia Willd. [Polygalaceae; Polygalae Radix] 10 g, Citrus medicaL.var. sarcodactylis Swingle. [Rutaceae; Citri Sarcodactμlis Fructus] 10 g	1 package bid po	Decoction	NR	NR
Tian et al., 2021	Jiaotai Pill	Hubei Provincial Hospital of Traditional Chinese Medicine	Partially reported^b^	Coptis chinensis Franch. [Ranunculaceae; Coptidis rhizoma], Neolitsea cassia (L.) Kosterm. [Lauraceae; Cinnamomi Cortex]	4 pills bid po	Pill	Yes	NR
Wang et al., 2016	Bushen Yiqi Huoxue Recipe	NR	NR	Astragalus mongholicus Bunge [Fabaceae; Astragali radix] 20g, Salvia miltiorrhiza Bunge [Lamiaceae; Salviae Miltiorrhizae Radix Et Rhizoma] 20g, Poria cocos (Schw.) Wolf [Polyporaceae; Poria] 15g, Panax ginseng C.A.Mey. [Araliaceae; Ginseng radix et rhizoma] 15g, Lycium barbarum L. [Solanaceae; Lycii fructus] 12 g, Rehmannig glutinosa Libosch. [Orobanchaceae; Rehmanniae radix praeparata] 15 g, Schisandra chinensis (Turcz.) Baill. [Schisandraceae; Schisandrae chinensis fructus] 8 g, Trichosanthes kirilowii Maxim. [Cucurbitaceae; Radix Trichosanthis] 10 g, Ophiopogon japonicus (Thunb.) Ker Gawl. [Asparagaceae; Ophiopogonis radix] 12 g, Dioscorea oppositifolia L. [Dioscoreaceae; Dioscoreae rhizoma] 10 g, Rubus chingii Hu [Rosaceae; Rubi Fructus] 12 g, Alisma plantago-aquatica L. [Alismataceae; Alismatis rhizoma] 12 g	1 package bid po	Decoction	NR	NR
Wang 2015	Bushen Jiannao Granules	NR	NR	Cistanche deserticola Ma [Orobanchaceae; Cistanches Herba] 15 g, Reynoutria multiflora (Thunb.) Moldenke [Polygonaceae; Polygoni multiflori radix] 15 g, Alpinia oxyphylla Miq. [Zingiberaceae; Alpiniae Oxyphyllae Fructus] 12 g, Rehmannia glutinosa (Gaertn.) DC. [Orobanchaceae; Rehmanniae radix praeparata] 15 g, Cornus officinalis Siebold & Zucc. [Cornaceae; Corni Fructus] 15 g, Dioscorea oppositifolia L. [Dioscoreaceae; Dioscoreae rhizoma] 15g, Polygala tenuifolia Willd. [Polygalaceae; Polygalae Radix] 12 g, Acorus calamus var. angustatus Besser [Acoraceae; Acori tatarinowii rhizoma] 10 g	150 ml bid po	Granule	NR	NR
Wang et al., 2015	Bushen Huoxue Decoction	NR	NR	Polygonatum sibiricum Redouté [Asparagaceae; Polygonati Rhizoma] 30 g, Rehmannig glutinosa Libosch. [Orobanchaceae; Rehmanniae radix praeparata] 15 g, Rehmannia glutinosa (Gaertn.) DC. [Orobanchaceae; Rehmanniae radix praeparata] 15 g, Cistanche deserticola Ma [Orobanchaceae; Cistanches Herba] 15 g, Astragalus mongholicus Bunge [Fabaceae; Astragali radix] 30 g, Panax quinquefolium L. [Araliaceae; Panacis Quinquefolii Radix] 10 g, Ginkgo biloba L. [Zingiberaceae; Ginkgo Folium] 15 g, Hirudo niponica Whitman [Hirudo; Hirudo] 2 g, Eupolyphaga sinensis Walker [Eupolyphaga; Eupolyphaga] 10 g, Alisma plantago-aquatica L. [Alismataceae; Alismatis rhizoma] 15 g, Acorus calamus var. angustatus Besser [Acoraceae; Acori tatarinowii rhizoma] 12 g, Polygala tenuifolia Willd. [Polygalaceae; Polygalae Radix] 10 g, Paeonia suffruticosa Andr. [Paeoniaceae; Moutan Cortex] 10 g, Actaea cimicifuga L. [Ranunculaceae; Cimicifugae rhizoma] 10 g, Glycyrrhiza uralensis Fisch. ex DC. [Fabaceae; Glycyrrhizae radix et rhizoma] 6 g	1 package bid po	Decoction	NR	NR
Yan and Guan 2019	Yizhi Mixture	Affiliated Hospital of Shandong University of Traditional Chinese Medicine	NR	Rehmannia glutinosa (Gaertn.) DC. [Orobanchaceae; Rehmanniae radix praeparata] 30 g, Gynochthodes officinali How [Rubiaceae; Morindae Officinalis Radix] 30 g, Codonopsis pilosula (Franch.) Nannf. [Campanulaceae; Codonopsis radix] 12 g, Ophiopogon japonicus (Thunb.) Ker Gawl. [Asparagaceae; Ophiopogonis radix] 15 g, Cuscuta chinensis Lam. [Convolvulaceae; Cuscutae Semen] 30 g, Ziziphus jujuba Mill.var.spinosa (Bunge) Hu ex H.F.Chou [Rhamnaceae; Ziziphi Spinosae Semen] 30 g, Polygala tenuifolia Willd. [Polygalaceae; Polygalae Radix] 6 g, Bupleurum Chinense DC. [Apiaceae; Bupleuri radix] 3 g, Paeonia lactiflora Pall. [Paeoniaceae; Paeoniae Radix Alba] 15 g, Poria cocos (Schw.) Wolf [Polyporaceae; Poria] 5 g, Salvia miltiorrhiza Bunge [Lamiaceae; Salviae Miltiorrhizae Radix Et Rhizoma] 9 g, Glycyrrhiza uralensis Fisch. ex DC. [Fabaceae; Glycyrrhizae radix et rhizoma] 3 g	50 ml bid po	Decoction	NR	NR
Yang 2017	Shenqi Yizhi Jiannao Recipe	Jiangsu Province Hospital of TCM	Partially reported^c^	Pseudostellaria heterophylla (Miq.)Pax ex Paxet Hoffm. [Caryophyllaceae; Pseudostellariae Radix] 10 g, Astragalus mongholicus Bunge [Fabaceae; Astragali radix] 10 g, Poria cocos (Schw.) Wolf [Polyporaceae; Poria] 10 g, Rehmannia glutinosa (Gaertn.) DC. [Orobanchaceae; Rehmanniae radix praeparata] 10g, Cornus officinalis Siebold & Zucc. [Cornaceae; Corni Fructus] 10 g, Panax notoginseng (Burkill) F.H.Chen [Araliaceae; Notoginseng radix et rhizoma] 5 g, Acorus calamus var. angustatus Besser [Acoraceae; Acori tatarinowii rhizoma] 10 g, Polygala tenuifolia Willd. [Polygalaceae; Polygalae Radix] 6 g, Alpinia oxyphylla Miq. [Zingiberaceae; Alpiniae Oxyphyllae Fructus] 10 g	NR	Decoction	NR	NR
Yu 2018	Xuefu Zhuyu Decoction	NR	NR	Prunus persica (L.) Batsch [Rosaceae; Persicae semen] 15 g, Carthamus tinctorius L. [Asteraceae; Carthami Flos] 12 g, Angelica sinensis (Oliv.) Diels [Apiaceae; Angelicae Sinensis Radix] 15 g, Ligusticum chuanxiong Hort. [Apiaceae; Chuanxiong rhizoma] 12 g, Paeonia veitchii Lynch. [Paeoniaceae; Paeoniae Radix Rubra] 15 g, Rehmannig glutinosa Libosch. [Orobanchaceae; Rehmanniae radix praeparata] 15 g, Bupleurum Chinense DC. [Apiaceae; Bupleuri radix] 15 g, Paeonia lactiflora Pall. [Paeoniaceae; Paeoniae Radix Alba] 12 g, Citrus aurantium L. [Rutaceae; Fructus Aurantii] 12 g, Glycyrrhiza uralensis Fisch. ex DC. [Fabaceae; Glycyrrhizae radix et rhizoma] 10 g, Achyranthes bidentata Blume [Amaranthaceae; Radix Achyranthis Bidentatae ] 15 g, Astragalus mongholicus Bunge [Fabaceae; Astragali radix] 20 g	200 ml bid po	Decoction	NR	NR
Yu et al., 2022	Bushen Jianpi Huoxue Recipe	NR	Partially reported^c^	Rehmannia glutinosa (Gaertn.) DC. [Orobanchaceae; Rehmanniae radix praeparata] 15g, Lycium barbarum L. [Solanaceae; Lycii fructus] 15g, Achyranthes bidentata Blume [Amaranthaceae; Radix Achyranthis Bidentatae ] 15g, Astragalus mongholicus Bunge [Fabaceae; Astragali radix] 20g, Codonopsis pilosula (Franch.) Nannf. [Campanulaceae; Codonopsis radix] 15g, Atractylodes macrocephala Koidz. [Asteraceae; Atractylodis macrocephalae rhizoma] 15g, Angelica sinensis (Oliv.) Diels [Apiaceae; Angelicae Sinensis Radix] 15g, Prunus persica (L.) Batsch [Rosaceae; Persicae semen] 15g, Carthamus tinctorius L. [Asteraceae; Carthami Flos] 12g, Acorus calamus var. angustatus Besser [Acoraceae; Acori tatarinowii rhizoma] 15g, Glycyrrhiza uralensis Fisch. ex DC. [Fabaceae; Glycyrrhizae radix et rhizoma] 6 g	250 ml bid po	Decoction	NR	NR
Zhang 2016	Yizhi Mixture	Affiliated Hospital of Shandong University of Traditional Chinese Medicine	Partially reported^c^	Rehmannia glutinosa (Gaertn.) DC. [Orobanchaceae; Rehmanniae radix praeparata] 150g, Ophiopogon japonicus (Thunb.) Ker Gawl. [Asparagaceae; Ophiopogonis radix] 75g, Ziziphus jujuba Mill.var.spinosa (Bunge) Hu ex H.F.Chou [Rhamnaceae; Ziziphi Spinosae Semen] 150g, Polygala tenuifolia Willd. [Polygalaceae; Polygalae Radix] 30g, Gynochthodes officinali How [Rubiaceae; Morindae Officinalis Radix] 150g, Cuscuta chinensis Lam. [Convolvulaceae; Cuscutae Semen] 150g, Codonopsis pilosula (Franch.) Nannf. [Campanulaceae; Codonopsis radix] 60g, Bupleurum Chinense DC. [Apiaceae; Bupleuri radix] 15g, Paeonia lactiflora Pall. [Paeoniaceae; Paeoniae Radix Alba] 75g, Poria cocos (Schw.) Wolf [Polyporaceae; Poria] 75g, Salvia miltiorrhiza Bunge [Lamiaceae; Salviae Miltiorrhizae Radix Et Rhizoma] 60g, Glycyrrhiza uralensis Fisch. ex DC. [Fabaceae; Glycyrrhizae radix et rhizoma] 15 g	50 ml bid po	Decoction	NR	NR
Zhao et al., 2014	Bushen Huoxue Kaiqiao Recipe	NR	NR	Cistanche deserticola Ma [Orobanchaceae; Cistanches Herba] 10g, Acorus calamus var. angustatus Besser [Acoraceae; Acori tatarinowii rhizoma] 5g, Panax notoginseng (Burkill) F.H.Chen [Araliaceae; Notoginseng radix et rhizoma] 2.5 g	1 package tid po	Decocted preparation	NR	NR

NR, not reported; bid, twice a day; tid, three times a day.

^a^ Extraction temperature and time and amount of the provoked extract were reported but not the amount of the initial solvent.

^b^ Only the amount of provoked extract was reported.

^c^ Referred to simply as “boiling.”

**FIGURE 2:**
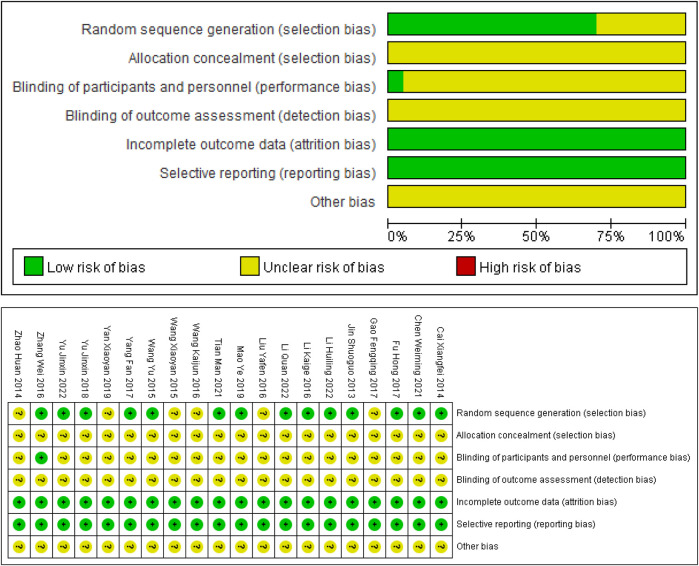
Included studies’ risk of bias plot.

Assessment of risk biases were outlined as Figure 2. In general, the overall methodological qualities of the included studies were poor to moderate. In terms of random sequence generation, 14 studies provided a sufficient randomization process to generate random sequences with a low risk of bias, whereas the remaining 6 studies supplied unspecific details of randomization, and thus were assessed as unclear risk. None of the included studies explicitly mentioned the use of allocation concealment, which led to unclear risk of bias in the relative domain. Only 1 study described the implementation of single blinding of subjects, which was rated as low risk. None of other included studies explicitly mentioned the use of blind method, resulting in an unclear associated risk of bias. All studies included in the analysis published complete data regarding the outcomes, leading us to rate the risk of bias as low. Meanwhile, all included RCTs didn’t report the bias of selective reporting, thus assessing as low risk. As for other biases, none of the studies provided adequate information for risk judgment, resulting in an unclear risk of bias.

### 3.3 Meta-analysis results

#### 3.3.1 Meta-analysis of total effective rate

**FIGURE 3:**
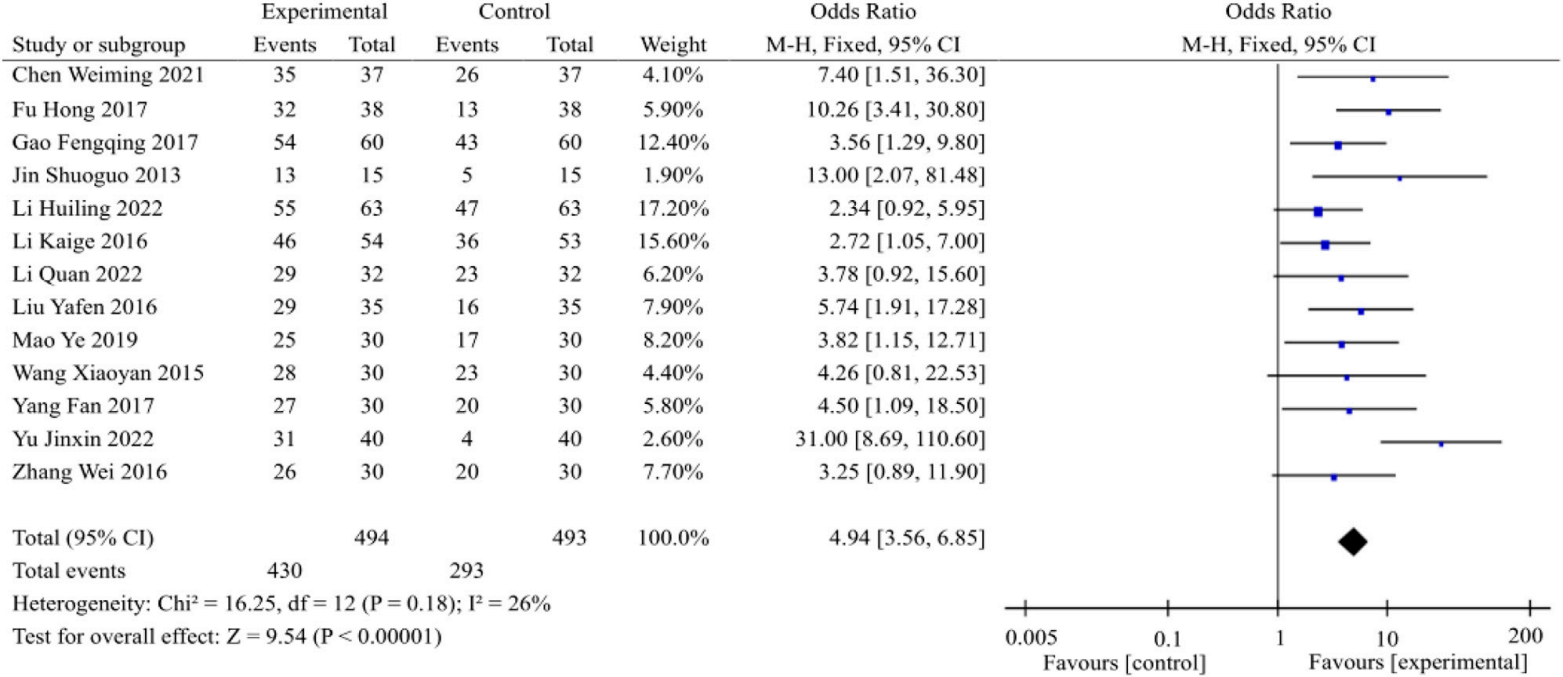
Total effective rate meta-analysis forest plot.

Preliminary data from 13 studies revealed the disclosure of the total effective rate, as illustrated in Figure 3. Following the heterogeneity test (*p* = 0.18, I^2^ = 26%), the fixed-effects model was employed for analysis. The findings demonstrated a statistically significant increase in the total effective rate within the experimental group compared to the control group [OR = 4.94, 95% CI (3.56, 6.85), *p* < 0.00001]. Consequently, the combination of TCM and WM exhibited superior efficacy in treating DACD when compared to WM alone.

An additional subgroup analysis revealed that the combination of TCM and WM resulted in a significantly higher total effective rate compared to WM alone for treatment durations of 2 months [*p* = 0.77, I^2^ = 0%; OR = 3.01, 95% CI = (1.57, 5.77), *p* = 0.0009], 3 months [*p* = 0.16, I^2^ = 36%; OR = 7.47, 95% CI = (4.50, 12.41), *p* < 0.00001], and 6 months [*p* = 0.41, I^2^ = 0%; OR = 4.17, 95% CI (2.31, 7.51), *p* < 0.00001].

#### 3.3.2 Meta-analysis of FPG

**FIGURE 4:**
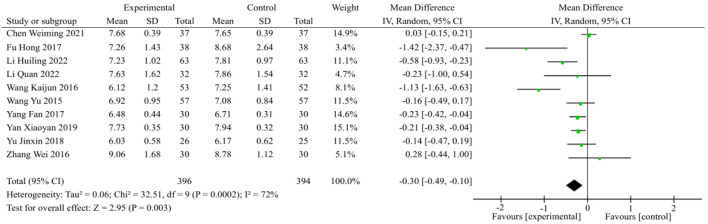
FPG meta-analysis forest plot.

A total of 10 studies were incorporated into the analysis of FPG, as illustrated in Figure 4. The random effects model was chosen after considering the outcomes of the heterogeneity test (*p* = 0.0002, I^2^ = 72%). In comparison to the control group, the combination of TCM and WM demonstrated a significant reduction in FPG levels among patients with DACD [MD = −0.30, 95% CI (−0.49, −0.10), *p* = 0.003].

A subgroup analysis indicated not readily apparent heterogeneity (*p* = 0.19, I^2^ = 40%) during the ≤2 month treatment course. On the basis of the fixed effects model, TCM plus WM treatment of DACD was determined to induce a statistically significant effect compared with WM alone [MD = −0.34, 95%CI (−0.57, −0.11), *p* = 0.004]. Regarding the 3-month treatment course (*p* = 0.0006, I^2^ = 79%) and 6-month treatment course (*p* = 0.06, I^2^ = 73%), heterogeneity was significant, and the statistical analysis using the random effects model indicated that TCM plus WM was superior to the control group in terms of reducing FPG level during the 3-month treatment course [MD = −0.31, 95%CI (−0.46, −0.15), *p* < 0.0001]. However, there was no statistical significance between the two groups during 6-month treatment course [MD = −0.10, 95% CI (−0.22, 0.03), *p* = 0.13].

#### 3.3.3 Meta-analysis of HbA1c

**FIGURE 5:**
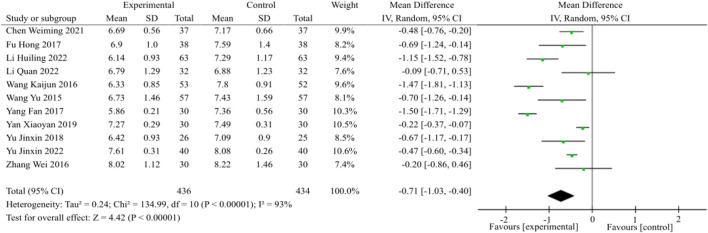
HbA1c meta-analysis forest plot. The subgroup analysis revealed that the benefits of TCM plus WM on lowering HbA1c surpassed that of the control group, irrespective of whether the duration was less than or equal to 2 months [*p* = 0.01, I^2^ = 77%; MD = −0.68, 95%CI (−1.27, −0.09), *p* = 0.02], 3 months [*p* < 0.00001, I^2^ = 94%; MD = −0.86, 95%CI (−1.38, −0.35), *p* = 0.001] or 6 months [*p* = 0.11, I^2^ = 61%; MD = −0.32, 95%CI (−0.57, −0.07), *p* = 0.01].

Eleven studies involving a total of 870 patients were included in the analysis, as illustrated in Figure 5. The statistical analysis was conducted using the random effects model, following the heterogeneity test (*p* < 0.00001, I^2^ = 93%). The findings of the meta-analysis revealed that the combination of TCM and WM exhibited a greater capacity to decrease HbA1c levels compared to the use of WM alone [MD = −0.71, 95%CI (−1.03, −0.40), *p* < 0.00001].

#### 3.3.4 Meta-analysis of MoCA score

**FIGURE 6:**
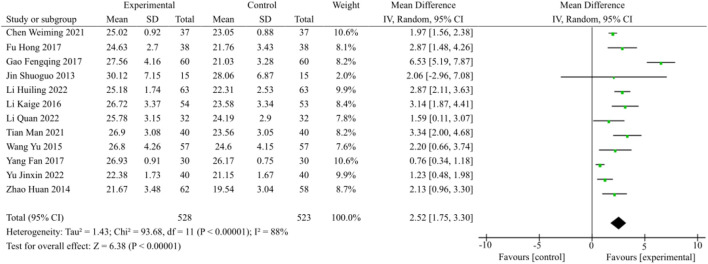
MoCA score meta-analysis forest plot.

12 studies focused on MoCA scores, as illustrated in Figure 6. The heterogeneity was apparent (*p* < 0.00001, I^2^ = 88%); Therefore, the random effects model was selected. In patients with DACD, TCM plus WM significantly improved MoCA scores compared with WM alone [MD = 2.52, 95% CI (1.75, 3.30), *p* < 0.00001].

The MoCA scale consisted of the following 7 items, including visuospatial and executive, naming, attention, language, abstraction, memory and delayed recall, as well as orientation. In the present study, a total of 5 studies reported on the specific items of MoCA scale in detail. The pooled data of meta-analysis demonstrated the superior effect of TCM plus WM for items other than naming and orientation. The entire summary was provided in Table 3.

**TABLE 3:** Meta-analysis of the 7 items of MoCA scale.

Outcomes	Meta-analysis results		Effect model	Heterogeneity test	
	MD (95%CI)	*p*-value		*p*-value	I^2^ (%)
Visuospatial and Executive	0.54 (0.15, 0.93)	0.007	Random	<0.00001	87
Naming	0.07 (−0.01, 0.14)	0.08	Fixed	0.38	5
Attention	0.57 (0.44, 0.69)	<0.00001	Fixed	0.52	0
Language	0.34 (0.24, 0.45)	<0.00001	Fixed	0.31	17
Abstraction	0.31 (0.07, 0.56)	0.01	Random	<0.00001	90
Memory and Delayed recall	0.39 (0.26, 0.52)	<0.00001	Fixed	0.21	32
Orientation	0.21 (−0.01, 0.43)	0.06	Random	<0.0001	85

#### 3.3.5 Meta-analysis of MMSE score

**FIGURE 7:**
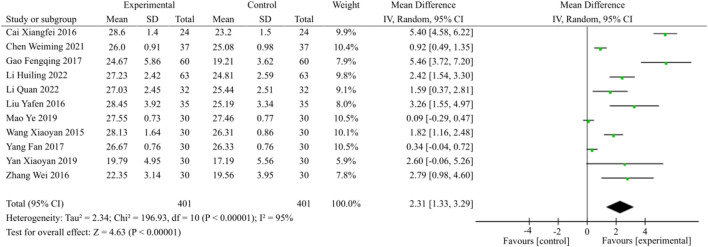
MMSE score meta-analysis forest plot.

A total of 11 eligible studies, as illustrated in Figure 7, encompassing 802 patients evenly distributed across two study groups, were included for the purpose of analyzing the MMSE score. The random effects model was chosen for analysis due to the observed heterogeneity (*p* < 0.00001, I^2^ = 95%). The combination of TCM and WM demonstrated a significant improvement in MMSE scores among patients with DACD compared to the control group [MD = 2.31, 95% CI (1.33, 3.29), *p* < 0.00001].

#### 3.3.6 Meta-analysis of TNF-α

**FIGURE 8:**
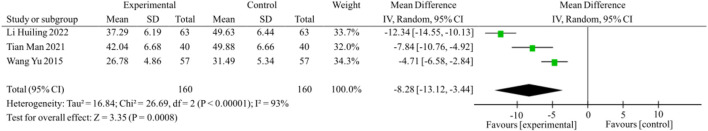
TNF-α meta-analysis forest plot.

Furthermore, three studies specifically focused on the reduction of TNF-α, as illustrated in Figure 8. The random effects model was employed for analysis based on the results of the heterogeneity test (*p* < 0.00001, I^2^ = 93%). The meta-analysis results indicated a statistically significant difference between the two groups [MD = −8.28, 95%CI (−13.12, −3.44), *p* = 0.0008]. The findings of this study suggest that the combination of TCM and WM in the treatment of DACD is more effective in reducing TNF-α levels compared to WM alone (Figure 5).

#### 3.3.7 Meta-analysis of TCM syndrome score

**FIGURE 9:**
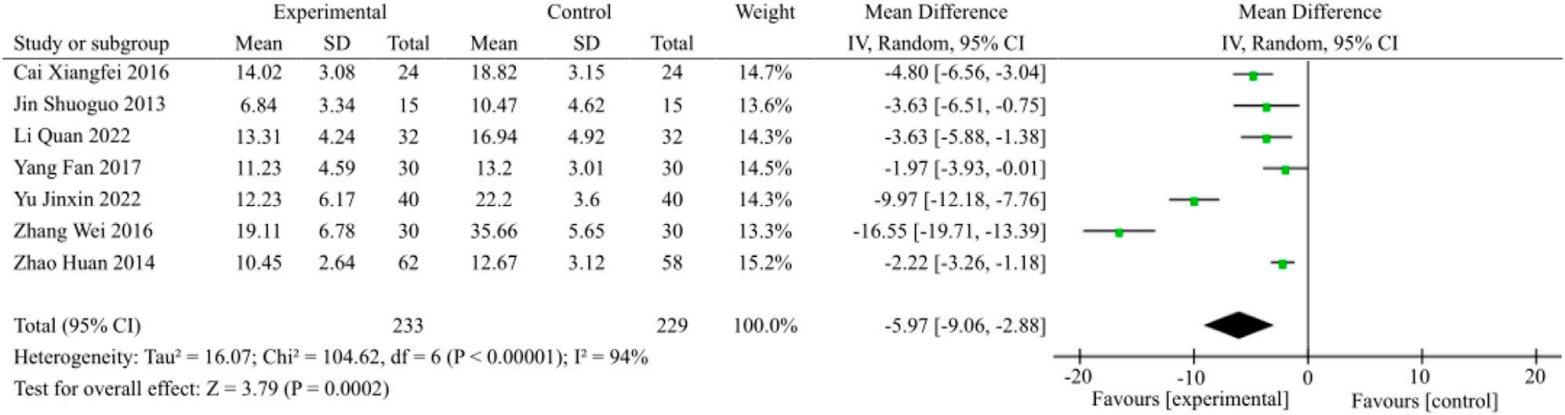
TCM syndrome score meta-analysis forest plot.

Out of the included studies, a total of 7 articles reported improvements in TCM syndrome, as illustrated in Figure 9. Heterogeneity testing was conducted, followed by a meta-analysis using the random-effects model (*p* < 0.00001, I^2^ = 94%). The results showed a statistically significant difference with a MD of −5.97 and a 95% CI of (−9.06, −2.88). These findings indicate that the combination of TCM and WM is more effective in treating DACD by improving TCM syndrome (*p* = 0.0002).

#### 3.3.8 Meta-analysis of adverse reactions

**FIGURE 10:**
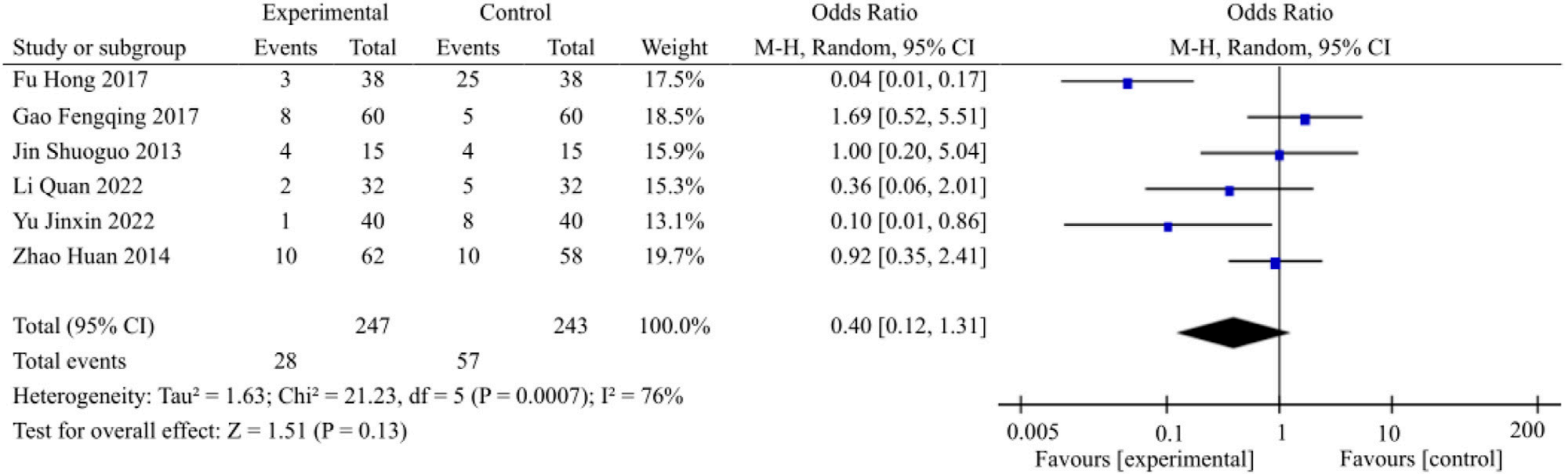
Adverse reaction meta-analysis forest plot.

Twelve studies within the encompassed literature made reference to the occurrence of adverse reactions, with only six of these studies reporting patients who experienced such reactions. Conversely, the remaining six studies documented no instances of adverse reactions among their respective patient populations. A total of 28 patients in the experimental group had adverse reactions during treatment, including 7 cases of dizziness, 2 cases of headache, 2 cases of gastrointestinal discomfort, 2 cases of facial flushing, 5 cases of nausea and vomiting, 4 cases of diarrhea, 1 case of albuminuria, 1 case of xerostomia, 1 case of fever, 2 cases of restlessness, and 1 case of insomnia; A total of 57 patients in the control group experienced adverse reactions, including 11 cases of dizziness, 7 cases of headache, 10 cases of gastrointestinal discomfort, 6 cases of decreased blood pressure, 1 case of facial flushing, 3 cases of nausea and vomiting, 1 case of diarrhea, 1 case of anemia, 2 cases of albuminuria, 1 case of impaired liver function, 2 cases of impaired renal function, 2 cases of xerostomia, 2 cases of fever, 2 cases of restlessness, 2 cases of insomnia, 2 cases of tinnitus, and 2 cases of constipation. The overall heterogeneity was manifested (*p* = 0.007, I^2^ = 76%), and thus the random effects model was selected. The data of meta-analysis showed that the differences were not statistically significant (OR = 0.40, 95% CI [0.12, 1.31], *p* = 0.13) (Figure 10). This observation suggests that the safety of medication administration was comparable between the experimental and control groups, namely, on the basis of WM, the additional use of TCM didn’t appear to result in an increase in adverse reactions.

### 3.4 Sensitivity analysis and publication bias

In order to conduct a sensitivity analysis on the total effective rate, FPG, HbA1c, MoCA score, MMSE score, TCM syndrome score, TNF-α, and adverse reactions, a meticulous item-by-item elimination approach was employed to scrutinize the data extracted from the included literature. No notable alterations were observed in the stability of each study and the combined outcomes of each effect size, thereby affirming the credibility of the data analysis findings. Additionally, Egger’s test was conducted for each outcome to evaluate the likelihood of publication bias, with a significance level of *p* < 0.05 indicating the presence of such bias. The examination disclosed the absence of publication bias across all indicators. A comprehensive review of the literature indicated that all studies were conducted in China and reported favorable results. These details are comprehensively presented in Table 4. Therefore, it was postulated that the presence of publication bias could potentially be associated with geographical location, racial demographics, and the non-publication of negative findings.

**TABLE 4:** Summary of sensitivity analysis and publication bias.

Indicators	OR/MD fluctuations	95%CI fluctuations	Publication bias (*p*-value)	
			Begg’s test	Egger’s test
Total effective rate	4.94	(3.56, 6.85)	0.077	0.081
FPG	−0.37	(−0.59, −0.15)	0.721	0.498
HbA1c	−0.99	(−1.43, −0.57)	0.533	0.160
MoCA score	0.99	(0.71, 1.27)	0.837	0.781
MMSE score	0.97	(0.62, 1.32)	1.000	0.141
TCM syndrome score	−1.28	(−1.82, −0.74)	0.213	0.213
TNF-α	−1.35	(−1.98, −0.72)	1.000	0.825
Adverse reaction	0.42	(0.26, 0.69)	0.260	0.366

### 3.5 Evidence quality rating of outcome indicators

The GRADE pro software was employed to assess the quality of evidence. The primary outcome measure, total effective rate, demonstrated a moderate level of reliability, whereas the majority of outcome indicators were deemed to be of low-quality evidence. In addition, two outcome indicators, including TNF-α and AD, were graded as very low-quality evidence (Table 5).

**TABLE 5:** GRADE evidence quality of outcomes included in the literature.

Quality assessment							No. of patients		Effect		Quality	Importance
No. of studies	Design	Risk of bias	Inconsistency	Indirectness	Imprecision	Other considerations	Experimental	Control	Relative	Absolute		
Total Effective Rate												
13	RCT	Serious^a^	No serious inconsistency	No serious indirectness	No serious imprecision	None	430/494 (87%)	293/493 (59.4%)	OR 4.94 (3.56–6.85)	284 more 1,000 (from 245 more to 315 more)	Moderate	Critical
								66.7%		241 more per 1,000 (from 210 more to 265 more)		
FPG												
10	RCT	Serious^a^	Serious^b^	No serious indirectness	No serious imprecision	None	396	394	-	MD 0.3 lower (0.49–0.1 lower)	Low	Important
HbA1c												
11	RCT	Serious^a^	Serious^b^	No serious indirectness	No serious imprecision	None	436	434	-	MD 0.71 lower (1.03–0.4 lower)	Low	Important
MoCA Score												
12	RCT	Serious^a^	Serious^b^	No serious indirectness	No serious imprecision	None	528	523	-	MD 2.52 higher (1.75–3.3 higher)	Low	Important
MMSE Score												
11	RCT	Serious^a^	Serious^b^	No serious indirectness	No serious imprecision	None	401	401	-	MD 2.31 higher (1.33–3.29 higher)	Low	Important
TNF-α												
3	RCT	Serious^a^	Serious^b^	No serious indirectness	Serious^c^ ^,^ ^d^	None	160	160	-	MD 8.28 lower (13.12–3.44 lower)	Very low	Important
TCM Syndrome Score												
7	RCT	Serious^a^	Serious^b^	No serious indirectness	No serious imprecision	None	233	229	-	MD 5.97 lower (9.06–2.88 lower)	Low	Important
Adverse Reaction												
6	RCT	Serious^a^	Serious^b^	No serious indirectness	Serious^c^	None	28/247 (11.3%)	57/243 (23.5%)	OR 0.4 (0.12–1.31)	125 fewer 1,000 (from 199 fewer to 52 more)	Very low	Important
								18.6%		102 more per 1,000 (from 159 fewer to 44 more)		

^a^ Some study randomization methods, allocation concealment, and blinding are not described.

^b^ Heterogeneity is significantly higher.

^c^ The 95% Cl crosses the invalid line.

^d^ Fewer included articles and observers.

## 4 Discussion

### 4.1 Summary of evidence

In recent times, there has been a significant surge in the worldwide prevalence of diabetes mellitus. Data from the World Health Organization has emphasized that the estimated prevalence of diabetes is currently approaching 463 million adults with projection rising to 700 million by 2,045 years (Sun et al., 2022). Concurrently, the cognitive decline linked to diabetes has emerged as a growing area of concern. Substantial evidence has substantiated that DM serves as a significant etiological factor for cognitive impairments, which may subsequently advance to dementia (Datusalia and Sharma, 2016). The epidemiological data have long shown that T2DM populations are at significantly higher risk for developing cognitive deficits and dementia compared with healthy individuals (Seto et al., 2015; Bello-Chavolla et al., 2019). Moreover, a meta-analysis of such studies comprising 25 studies found that the T2DM had an approximately 50% and 60% greater risk of developing DACD and dementia, respectively (Cukierman et al., 2005). However, there is currently no vaccine or specific medicine for DACD. Hence, it is of great significance to identify novel and better anti-DACD drugs and explore new therapeutic schemes.

The application of syndrome differentiation in treatment represents a distinctive feature of the theoretical framework of TCM, and it holds that “Xiao Ke,” the signs of which are recognized as common symptoms of diabetes mellitus in modern medicine, is caused by the interaction of multiple factors, including advanced age, poor diet, emotional and mental disorders, viscera deficiency, and the depletion of a long-term illness. Its pathogenesis is determined as dryness-heat due to deficiency of yin, further resulting in disorder of viscera function and insufficiency of Qi and blood, Yin and Yang, gradually developing the combination of blood stasis and phlegm turbidity that may obstruct the meridians of brain, and eventually leading to the onset or aggravate of “Xiao Ke” complicated with “Chi Dai,” the symptoms of which are considered as analogous to DACD in modern medicine (Yang et al., 2021).

With an apparent dissatisfaction with the effect of conventional treatments, a growing number of DACD sufferers are turning to alternatives medicine, particularly TCM or integrated TCM and WM. It is of note that the integrated TCM and WM therapy has been shown to be more effective than single conventional treatment in improving the effectiveness of DACD and alleviating the side effects (Cai, 2016; Chen et al., 2021; Fu et al., 2017; Gao et al., 2017; Jin et al., 2015; Li, 2022; Li, 2016; Li et al., 2022; Liu et al., 2016; Mao et al., 2019; Tian et al., 2021; Wang et al., 2016; Wang, 2015; Wang et al., 2015; Yan and Guan, 2019; Yang, 2017; Yu, 2018; Yu et al., 2022; Zhang, 2016; Zhao et al., 2014). To date, integrated TCM and WM therapy for DACD has been reported increasingly, conversely, high-quality meta-analysis remains scarce. Here, we aimed to assess the efficacy and safety of integrated TCM and WM therapy for DACD and provide high quality evidence for its clinical therapeutic effects in this context.

The present meta-analysis incorporated 20 RCTs that met the inclusion criteria and included 1,570 subjects in total. In terms of total effective rate, the meta-analysis indicates that integrated TCM and WM therapy for DACD was superior to that of WM alone, possibly by improving glucose toxicity, membrane signal disorder, homeostasis imbalance, inflammation, oxidative stress injury and vascular diseases (Fiatarone Singh et al., 2014; Tian et al., 2016). Meanwhile, the combination therapy could significantly alleviate the TCM syndrome scores. Moreover, prolonged elevation of blood glucose levels has been demonstrated to exert detrimental effects on cognitive abilities and cerebral morphology (Thal et al., 2012). In our investigation, we evaluated both HbA1c and FPG as a composite measure to aid in the assessment of glycemic control. We confirmed that compared with WM alone, integrated TCM and WM therapy exhibited advantages in improving fasting plasma glucose and HbA1c levels. In addition, MMSE and MoCA most probably reflected lesion-associated cognitive impairments (Dong et al., 2010). Thus, we selected MMSE and MoCA for assessing cognitive function in the present meta-analysis. Our results demonstrated that the integrated TCM and WM significantly enhanced the MMSE and MoCA scores in DACD patients, indicating that the combination therapy is beneficial for the improvement of cognitive function. It has been reported that inflammation is associated with the onset of T2DM and progression of its complications, especially DACD. TNF-a is a cytokine with pro-inflammatory properties that was related to both T2DM and cognitive decline (Chu, 2013; Khosravi et al., 2013; Martínez-Mármol et al., 2019). Meta-analysis results showed that the combination therapy could lead to a mean greater reduction in TNF-α level, with statistically significant. Regarding safety, it suggested that the safety of integrated TCM and WM therapy was comparable to that of the WM alone.

### 4.2 Advantages and limitations

To our knowledge, this is the first meta-analysis to quantitatively estimate the beneficial effect of the integration of TCM and WM therapy for DACD, providing better evidence-based evaluation for the domestic and international researchers to understand this issue. Following the Cochrane Collaboration’s guidelines, the objective is to derive more comprehensive conclusions. The inclusion of additional outcome measures, like FPG, HbA1c, MoCA score, MMSE score, TNF-α, and TCM syndrome score, enables a multidimensional and multilevel assessment of the effectiveness of integrating traditional Chinese medicine (TCM) and Western medicine (WM) for treating DACD.

Despite our critical evaluation of the currently available evidence, some potential limitations should not be ignored. First and foremost, the methodological quality of our included studies was generally poor. The majority of the included trials were rated as “moderate” or “high” bias risk due to insufficient information on randomization process, allocation concealment, and methods of blinding outcome assessors. In particular, double-blinding was not conducted in all of the included studies. When both herbal decoction and WM are used as interventions, it is difficult to perform blinding due to their differences in external characteristics. Moreover, the review found that protocol registration information, quality control and chemical profile were not reported in any of the included studies, which has led to controversial conclusions. Second, despite the fact that sensitivity analysis verified the reliability of our findings, there was substantial heterogeneity in several of them. The clinical diagnosis and treatment of TCM rely on a comprehensive assessment of various factors, including the patient’s symptoms, tongue image, and pulse image, known as syndrome differentiation. Subsequently, treatments are tailored based on the identified TCM syndrome type, indicating that patients with distinct syndromes receive different medications, potentially resulting in heterogeneity. Additionally, the variability in composition and dosage of Chinese herbal medicine, along with diverse outcome indicators, may contribute to further heterogeneity in the study results. Furthermore, the presence of diverse western medicine interventions utilized in the control group constitutes an inherent factor contributing to the observed heterogeneity. Third, it is noteworthy that the studies encompassed within this research were exclusively conducted in China and published solely in the Chinese language. Consequently, the validation of our findings becomes imperative in order to ascertain the applicability of TCM in broader samples, diverse countries, and various ethnic groups. Fourth, Chinese clinical trials have become a relatively standardized, safe procedure in the last decade, and thus strict temporal constraints of the present study was made to uphold the study’s quality standards. However, this selection process led to the exclusion of a considerable number of studies that did not meet the intervention or diagnostic criteria. Thereafter, the insufficient number of included studies hindered the resolution of heterogeneities. Furthermore, a majority of the included studies had small sample sizes, which appeared to be one risk in exaggerating intervention benefits. Last but no the least, there is a dearth of studies that have presented follow-up data on the combination of TCM and WM in the treatment of DACD. Given the chronic and progressive nature of DACD, characterized by potential fluctuations over an extended period, it is imperative to conduct ongoing follow-up assessments to determine the true efficacy and long-term consequences of this therapeutic approach. Regrettably, the majority of studies have been limited by short treatment durations, with none of them encompassing long-term follow-up observations.

### 4.3 Implications for clinical practice and future research

Over the past dozen years, the integration of TCM and WM therapy is being increasingly investigated for their use in the treatment of DACD. However, the choice of Chinese herbal medicine is empirical and there is a lack of consensus among clinicians. The evidence available from our study demonstrated the effectiveness and safety of CHM therapy for DACD, which is able to offer a comprehensive and transparent framework for promoting clinical practice guidelines. Importantly, extensive and in-depth data mining in applying TCM to the treatment of DACD is conducive to inherit the clinical experience of ancient TCM, boost the understanding of the theory and practice of TCM in DACD treatment and enrich the treatment options for treating DACD. The findings from our study provide evidence supporting the efficacy and safety of the above-mentioned therapy for DACD, thereby offering a comprehensive and transparent framework for the promotion of clinical practice guidelines. Notably, the extensive and thorough study in the application of TCM for DACD treatment facilitates the preservation of ancient TCM clinical experience, enhances the comprehension of TCM theory and practice in DACD treatment, and expands the range of treatment options available for DACD management.

In light of our findings and aforementioned limitations, several recommendations can be made for future research and clinical practice. Firstly, it is recommended that multi-center studies with larger sample sizes be undertaken in order to augment the representativeness and reliability of the findings. Secondly, the efficacy of Traditional Chinese Medicine (TCM) hinges upon the precise differentiation and treatment of the syndrome, thus it is advisable to establish an evaluation system for assessing therapeutic effects that aligns with the distinctive attributes of TCM, and to explore pragmatic and discerning indicators for TCM. Thirdly, investigations pertaining to TCM ought to enhance their protocols and prioritize quality control, with particular emphasis on the meticulous implementation of randomization, blinding, and allocation concealment. We advise designing and reporting RCTs of DACD strictly according to the CONSORT 2010 statement (Schulz et al., 2010) and the CONSORT Extension for Chinese Herbal Medicine Formulas 2017 (Cheng et al., 2017). Fourthly, incorporating an appropriate follow-up period that aligns with the characteristics of the disease, in order to conduct longer-term research and clinical trials to confirm the long-term safety of TCM on DACD, explore the optimal dosage, duration of treatment, and potential adverse events, thereby providing valuable clinical insights. Fifthly, considering the research prospects associated with Chinese herbal medicines in the treatment of diabetes and its diverse complications, clinical and animal experimental studies focusing on the active ingredients of Chinese herbal medicines may elucidate the specific effects and intrinsic mechanisms of treating DACD, which could increase the evidence base for the clinical application of TCM in DACD as well as promote the inclusion of TCM in relevant international guidelines.

## 5 Conclusion

This systematic review has found some promising evidence for the integration of TCM and WM therapy in the treatment of DACD. Compared to WM alone, the integration of TCM and WM therapy appears to show a more favorable therapeutic effect. Meanwhile, it holds great potential in alleviating blood glucose, improving cognitive function, and reducing inflammation.

However, it is crucial to consider the limitations to the evidence base, notably the low methodological quality and small sample sizes of the included studies. These factors highlight the need for further research into the use of TCM for DACD. Specifically, we urgently need high-quality, multi-center studies with larger samples to strengthen the evidence supporting the clinical use of TCM for DACD. This kind of robust research is essential to guide clinical decision-making and to optimize patient care in the future.
