# Supplementary material for: Efficacy and safety of the integration of traditional Chinese medicine and western medicine in the treatment of diabetes-associated cognitive decline: a systematic review and meta-analysis
Source: Front Pharmacol. 2023 Nov 22;14:1280736. doi: 10.3389/fphar.2023.1280736 (PMC10703163; doi:10.3389/fphar.2023.1280736)

Supplementary File-Results of subgroup analysis

Subgroup analysis of the total clinical effective rate according to the treatment course

Study or Subgroup	Experimental Events	Experimental Total	Control Events	Control Total	Weight	Odds Ratio M-H, Fixed, 95% CI
**1.11.1 2 months**						
Li Huiling 2022	55	63	47	63	17.2%	2.34 [0.92, 5.95]
Li Quan 2022	29	32	23	32	6.2%	3.78 [0.92, 15.60]
Mao Ye 2019	25	30	17	30	8.2%	3.82 [1.15, 12.71]
**Subtotal (95% CI)**		**125**		**125**	**31.7%**	**3.01 [1.57, 5.77]**
Total events	109		87			
Heterogeneity: Chi² = 0.53, df = 2 (P = 0.77); I² = 0%						
Test for overall effect: Z = 3.31 (P = 0.0009)						
**1.11.2 3 months**						
Fu Hong 2017	32	38	13	38	5.9%	10.26 [3.41, 30.80]
Liu Yafen 2016	29	35	16	35	7.9%	5.74 [1.91, 17.28]
Wang Xiaoyan 2015	28	30	23	30	4.4%	4.26 [0.81, 22.53]
Yang Fan 2017	27	30	20	30	5.8%	4.50 [1.09, 18.50]
Yu Jinxin 2022	31	40	4	40	2.6%	31.00 [8.69, 110.60]
Zhang Wei 2016	26	30	20	30	7.7%	3.25 [0.89, 11.90]
**Subtotal (95% CI)**		**203**		**203**	**34.4%**	**7.47 [4.50, 12.41]**
Total events	173		96			
Heterogeneity: Chi² = 7.86, df = 5 (P = 0.16); I² = 36%						
Test for overall effect: Z = 7.78 (P < 0.00001)						
**1.11.3 6 months**						
Chen Weiming 2021	35	37	26	37	4.1%	7.40 [1.51, 36.30]
Gao Fengqing 2017	54	60	43	60	12.4%	3.56 [1.29, 9.80]
Jin Shuoguo 2013	13	15	5	15	1.9%	13.00 [2.07, 81.48]
Li Kaige 2016	46	54	36	53	15.6%	2.72 [1.05, 7.00]
**Subtotal (95% CI)**		**166**		**165**	**34.0%**	**4.17 [2.31, 7.51]**
Total events	148		110			
Heterogeneity: Chi² = 2.86, df = 3 (P = 0.41); I² = 0%						
Test for overall effect: Z = 4.74 (P < 0.00001)						
**Total (95% CI)**		**494**		**493**	**100.0%**	**4.94 [3.56, 6.85]**
Total events	430		293			
Heterogeneity: Chi² = 16.25, df = 12 (P = 0.18); I² = 26%						
Test for overall effect: Z = 9.54 (P < 0.00001)						
Test for subgroup differences: Chi² = 5.11, df = 2 (P = 0.08), I² = 60.8%						

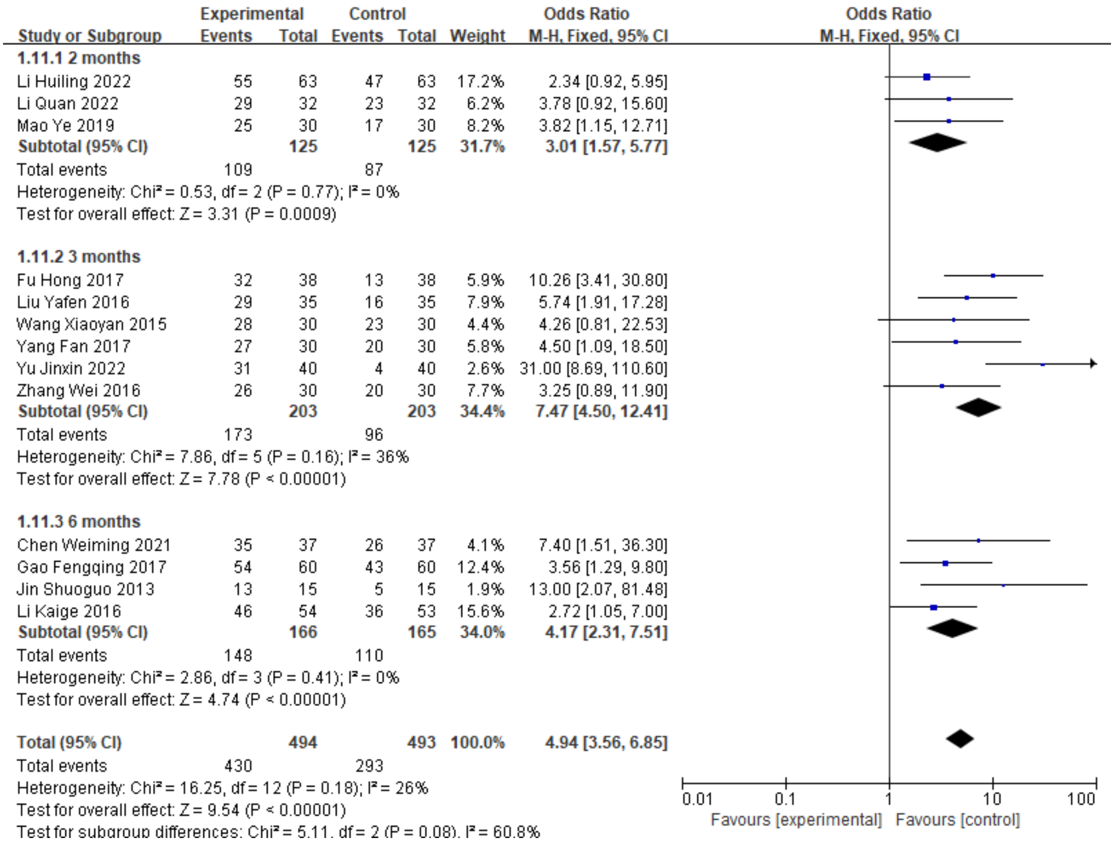

Subgroup analysis of the FPG level according to the treatment course

Study or Subgroup	Experimental Mean	Experimental SD	Experimental Total	Control Mean	Control SD	Control Total	Weight	Mean Difference IV, Random, 95% CI
**1.12.1 ⩽2 months**								
Li Huiling 2022	7.23	1.02	63	7.81	0.97	63	11.1%	-0.58 [-0.93, -0.23]
Li Quan 2022	7.63	1.62	32	7.86	1.54	32	4.7%	-0.23 [-1.00, 0.54]
Yu Jinxin 2018	6.03	0.58	26	6.17	0.62	25	11.5%	-0.14 [-0.47, 0.19]
**Subtotal (95% CI)**			**121**			**120**	**27.3%**	**-0.34 [-0.65, -0.02]**
Heterogeneity: $Tau^2 = 0.03; Chi^2 = 3.32, df = 2 (P = 0.19); I^2 = 40\%$								
Test for overall effect: Z = 2.08 (P = 0.04)								
**1.12.2 3 months**								
Fu Hong 2017	7.26	1.43	38	8.68	2.64	38	3.4%	-1.42 [-2.37, -0.47]
Wang Kaijun 2016	6.12	1.2	53	7.25	1.41	52	8.1%	-1.13 [-1.63, -0.63]
Wang Yu 2015	6.92	0.95	57	7.08	0.84	57	11.5%	-0.16 [-0.49, 0.17]
Yang Fan 2017	6.48	0.44	30	6.71	0.31	30	14.6%	-0.23 [-0.42, -0.04]
Zhang Wei 2016	9.06	1.68	30	8.78	1.12	30	5.1%	0.28 [-0.44, 1.00]
**Subtotal (95% CI)**			**208**			**207**	**42.7%**	**-0.46 [-0.89, -0.03]**
Heterogeneity: $Tau^2 = 0.17; Chi^2 = 19.49, df = 4 (P = 0.0006); I^2 = 79\%$								
Test for overall effect: Z = 2.09 (P = 0.04)								
**1.12.3 6 months**								
Chen Weiming 2021	7.68	0.39	37	7.65	0.39	37	14.9%	0.03 [-0.15, 0.21]
Yan Xiaoyan 2019	7.73	0.35	30	7.94	0.32	30	15.1%	-0.21 [-0.38, -0.04]
**Subtotal (95% CI)**			**67**			**67**	**30.0%**	**-0.09 [-0.33, 0.14]**
Heterogeneity: $Tau^2 = 0.02; Chi^2 = 3.66, df = 1 (P = 0.06); I^2 = 73\%$								
Test for overall effect: Z = 0.76 (P = 0.45)								
**Total (95% CI)**			**396**			**394**	**100.0%**	**-0.30 [-0.49, -0.10]**
Heterogeneity: $Tau^2 = 0.06; Chi^2 = 32.51, df = 9 (P = 0.0002); I^2 = 72\%$								
Test for overall effect: Z = 2.95 (P = 0.003)								
Test for subgroup differences: $Chi^2 = 2.85, df = 2 (P = 0.24), I^2 = 29.8\%$								

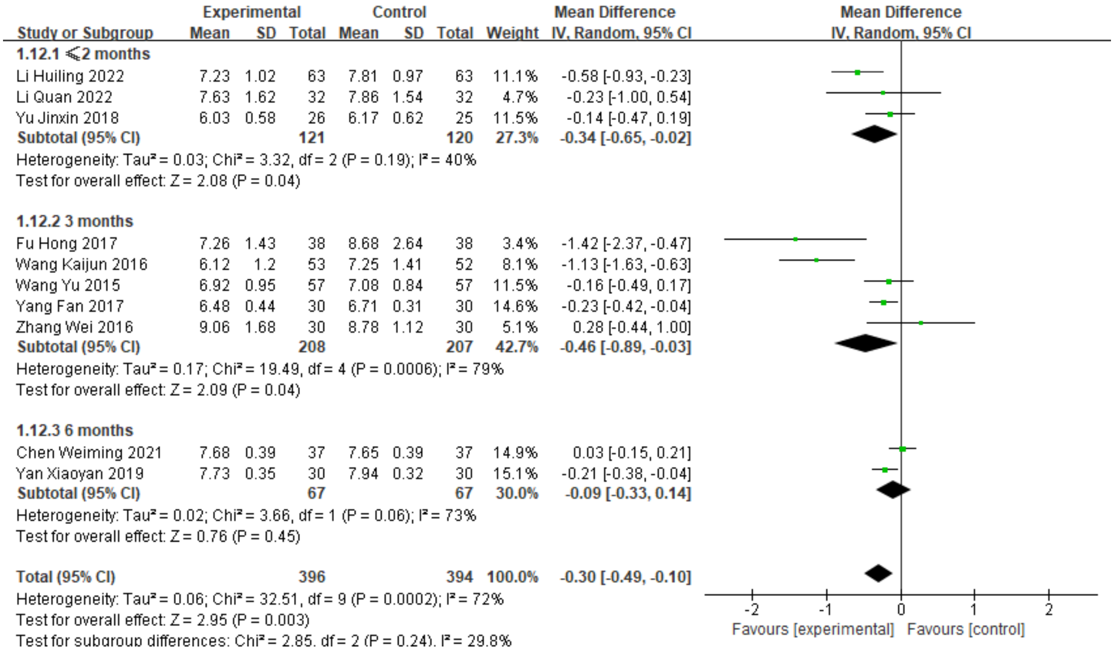

Subgroup analysis of the HbA1c level according to the treatment course

Study or Subgroup	Experimental Mean	Experimental SD	Experimental Total	Control Mean	Control SD	Control Total	Weight	Mean Difference IV, Random, 95% CI
**1.13.1 ≤2 months**								
Li Huiling 2022	6.14	0.93	63	7.29	1.17	63	9.4%	-1.15 [-1.52, -0.78]
Li Quan 2022	6.79	1.29	32	6.88	1.23	32	7.6%	-0.09 [-0.71, 0.53]
Yu Jinxin 2018	6.42	0.93	26	7.09	0.9	25	8.5%	-0.67 [-1.17, -0.17]
**Subtotal (95% CI)**			**121**			**120**	**25.5%**	**-0.68 [-1.27, -0.09]**
Heterogeneity: Tau² = 0.21; Chi² = 8.78, df = 2 (P = 0.01); I² = 77%								
Test for overall effect: Z = 2.24 (P = 0.02)								
**1.13.2 3 months**								
Fu Hong 2017	6.9	1	38	7.59	1.4	38	8.2%	-0.69 [-1.24, -0.14]
Wang Kaijun 2016	6.33	0.85	53	7.8	0.91	52	9.6%	-1.47 [-1.81, -1.13]
Wang Yu 2015	6.73	1.46	57	7.43	1.59	57	8.1%	-0.70 [-1.26, -0.14]
Yang Fan 2017	5.86	0.21	30	7.36	0.56	30	10.3%	-1.50 [-1.71, -1.29]
Yu Jinxin 2022	7.61	0.31	40	8.08	0.26	40	10.6%	-0.47 [-0.60, -0.34]
Zhang Wei 2016	8.02	1.12	30	8.22	1.46	30	7.4%	-0.20 [-0.86, 0.46]
**Subtotal (95% CI)**			**248**			**247**	**54.0%**	**-0.86 [-1.38, -0.35]**
Heterogeneity: Tau² = 0.36; Chi² = 86.23, df = 5 (P < 0.00001); I² = 94%								
Test for overall effect: Z = 3.30 (P = 0.0010)								
**1.13.3 6 months**								
Chen Weiming 2021	6.69	0.56	37	7.17	0.66	37	9.9%	-0.48 [-0.76, -0.20]
Yan Xiaoyan 2019	7.27	0.29	30	7.49	0.31	30	10.5%	-0.22 [-0.37, -0.07]
**Subtotal (95% CI)**			**67**			**67**	**20.5%**	**-0.32 [-0.57, -0.07]**
Heterogeneity: Tau² = 0.02; Chi² = 2.57, df = 1 (P = 0.11); I² = 61%								
Test for overall effect: Z = 2.54 (P = 0.01)								
**Total (95% CI)**			**436**			**434**	**100.0%**	**-0.71 [-1.03, -0.40]**
Heterogeneity: Tau² = 0.24; Chi² = 134.99, df = 10 (P < 0.00001); I² = 93%								
Test for overall effect: Z = 4.42 (P < 0.00001)								
Test for subgroup differences: Chi² = 4.07, df = 2 (P = 0.13), I² = 50.8%								

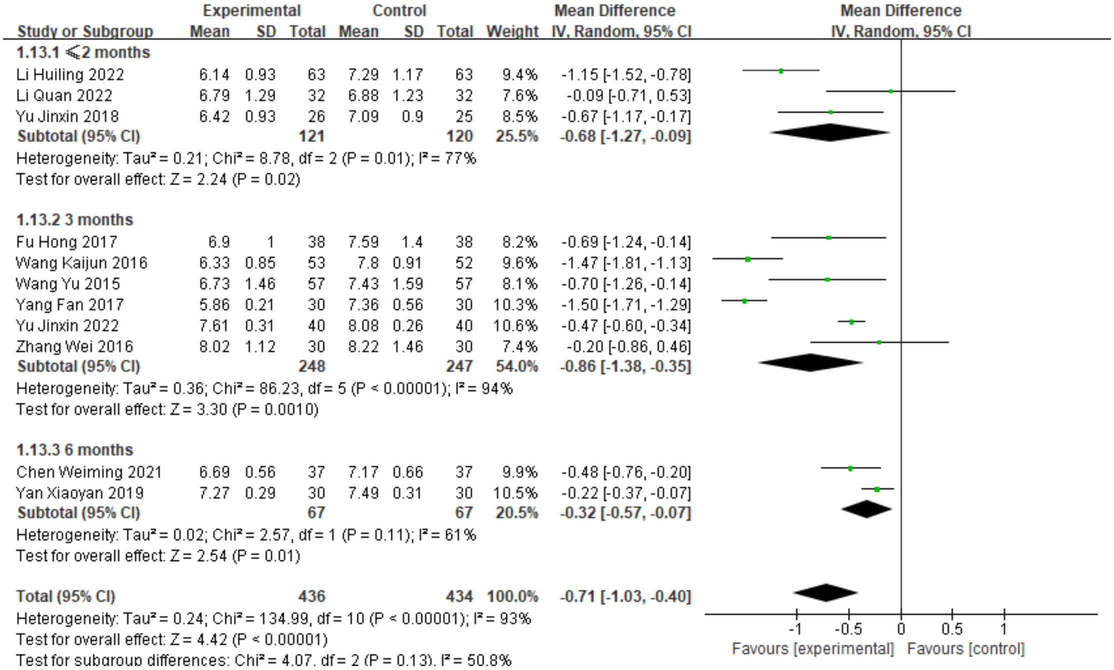

Subgroup analysis of the MoCA scale score according to the 7 different items

Study or Subgroup	Experimental Mean	Experimental SD	Experimental Total	Control Mean	Control SD	Control Total	Weight	Mean Difference IV, Random, 95% CI
**1.3.1 Visuospatial and Executive**								
Chen Weiming 2021	4.24	0.43	37	4.21	0.41	37	3.3%	0.03 [-0.16, 0.22]
Fu Hong 2017	3.74	1	38	3.13	1.02	38	1.9%	0.61 [0.16, 1.06]
Tian Man 2021	4.35	0.91	40	3.22	0.8	40	2.3%	1.13 [0.75, 1.51]
Wang Yu 2015	4.84	0.77	57	4.22	0.82	57	2.8%	0.62 [0.33, 0.91]
Zhao Huan 2014	3.31	0.9	62	2.92	1.03	58	2.4%	0.39 [0.04, 0.74]
**Subtotal (95% CI)**			**234**			**230**	**12.8%**	**0.54 [0.15, 0.93]**
Heterogeneity: Tau² = 0.17; Chi² = 31.75, df = 4 (P < 0.00001); I² = 87%								
Test for overall effect: Z = 2.69 (P = 0.007)								
**1.3.2 Naming**								
Chen Weiming 2021	2.97	0.16	37	2.94	0.22	37	3.8%	0.03 [-0.06, 0.12]
Fu Hong 2017	2.74	0.45	38	2.55	0.69	38	2.9%	0.19 [-0.07, 0.45]
Tian Man 2021	2.81	0.81	40	2.49	0.59	40	2.6%	0.32 [0.01, 0.63]
Wang Yu 2015	2.85	0.86	57	2.83	0.75	57	2.7%	0.02 [-0.28, 0.32]
Zhao Huan 2014	2.52	0.66	62	2.43	0.45	58	3.3%	0.09 [-0.11, 0.29]
**Subtotal (95% CI)**			**234**			**230**	**15.4%**	**0.07 [-0.01, 0.15]**
Heterogeneity: Tau² = 0.00; Chi² = 4.23, df = 4 (P = 0.38); I² = 5%								
Test for overall effect: Z = 1.75 (P = 0.08)								
**1.3.3 Attention**								
Chen Weiming 2021	4.45	0.55	37	3.89	0.56	37	3.0%	0.56 [0.31, 0.81]
Fu Hong 2017	5.55	0.6	38	5.21	0.99	38	2.3%	0.34 [-0.03, 0.71]
Tian Man 2021	5.51	1.1	40	5.12	0.68	40	2.2%	0.39 [-0.01, 0.79]
Wang Yu 2015	5.69	0.74	57	5.08	0.77	57	2.8%	0.61 [0.33, 0.89]
Zhao Huan 2014	5	0.56	62	4.33	0.62	58	3.2%	0.67 [0.46, 0.88]
**Subtotal (95% CI)**			**234**			**230**	**13.6%**	**0.57 [0.44, 0.69]**
Heterogeneity: Tau² = 0.00; Chi² = 3.21, df = 4 (P = 0.52); I² = 0%								
Test for overall effect: Z = 8.92 (P < 0.00001)								
**1.3.4 Language**								
Chen Weiming 2021	2.54	0.5	37	2.21	0.41	37	3.2%	0.33 [0.12, 0.54]
Fu Hong 2017	1.76	0.63	38	1.45	0.65	38	2.8%	0.31 [0.02, 0.60]
Tian Man 2021	1.8	0.67	40	1.59	0.77	40	2.6%	0.21 [-0.11, 0.53]
Wang Yu 2015	2.53	0.51	57	2.04	0.47	57	3.4%	0.49 [0.31, 0.67]
Zhao Huan 2014	1.78	0.75	62	1.6	0.7	58	2.9%	0.18 [-0.08, 0.44]
**Subtotal (95% CI)**			**234**			**230**	**15.0%**	**0.34 [0.22, 0.45]**
Heterogeneity: Tau² = 0.00; Chi² = 4.82, df = 4 (P = 0.31); I² = 17%								
Test for overall effect: Z = 5.62 (P < 0.00001)								
**1.3.5 Abstraction**								
Chen Weiming 2021	2	0.01	37	1.97	0.16	37	3.9%	0.03 [-0.02, 0.08]
Fu Hong 2017	1.45	0.72	38	0.63	0.71	38	2.6%	0.82 [0.50, 1.14]
Tian Man 2021	1.42	0.61	40	1.32	0.4	40	3.1%	0.10 [-0.13, 0.33]
Wang Yu 2015	1.78	0.46	57	1.52	0.44	57	3.5%	0.26 [0.09, 0.43]
Zhao Huan 2014	1.56	0.59	62	1.06	0.69	58	3.1%	0.50 [0.27, 0.73]
**Subtotal (95% CI)**			**234**			**230**	**16.3%**	**0.31 [0.07, 0.56]**
Heterogeneity: Tau² = 0.06; Chi² = 41.21, df = 4 (P < 0.00001); I² = 90%								
Test for overall effect: Z = 2.55 (P = 0.01)								
**1.3.6 Memory and Delayed recall**								
Chen Weiming 2021	3.32	0.52	37	3	0.52	37	3.1%	0.32 [0.08, 0.56]
Fu Hong 2017	3.58	1.24	38	2.55	1.48	38	1.3%	1.03 [0.42, 1.64]
Tian Man 2021	3.54	1.08	40	2.89	1.22	40	1.7%	0.65 [0.15, 1.15]
Wang Yu 2015	2.59	0.47	57	2.25	0.48	57	3.4%	0.34 [0.17, 0.51]
Zhao Huan 2014	2.11	1.67	62	1.65	1.67	58	1.4%	0.46 [-0.14, 1.06]
**Subtotal (95% CI)**			**234**			**230**	**11.0%**	**0.43 [0.25, 0.62]**
Heterogeneity: Tau² = 0.01; Chi² = 5.90, df = 4 (P = 0.21); I² = 32%								
Test for overall effect: Z = 4.60 (P < 0.00001)								
**1.3.7 Orientation**								
Chen Weiming 2021	4.91	0.27	37	4.86	0.34	37	3.6%	0.05 [-0.09, 0.19]
Fu Hong 2017	5.87	0.41	38	5.95	0.23	38	3.6%	-0.08 [-0.23, 0.07]
Tian Man 2021	6.8	0.52	40	6.22	0.51	40	3.1%	0.58 [0.35, 0.81]
Wang Yu 2015	6.85	0.99	57	6.43	1.08	57	2.3%	0.42 [0.04, 0.80]
Zhao Huan 2014	5.64	0.51	62	5.43	0.46	58	3.4%	0.21 [0.04, 0.38]
**Subtotal (95% CI)**			**234**			**230**	**16.0%**	**0.21 [-0.01, 0.43]**
Heterogeneity: Tau² = 0.05; Chi² = 27.16, df = 4 (P < 0.0001); I² = 85%								
Test for overall effect: Z = 1.88 (P = 0.06)								
**Total (95% CI)**			**1638**			**1610**	**100.0%**	**0.34 [0.25, 0.43]**
Heterogeneity: Tau² = 0.05; Chi² = 209.80, df = 34 (P < 0.00001); I² = 84%								
Test for overall effect: Z = 7.64 (P < 0.00001)								
Test for subgroup differences: Chi² = 52.34, df = 6 (P < 0.00001), I² = 88.5%								

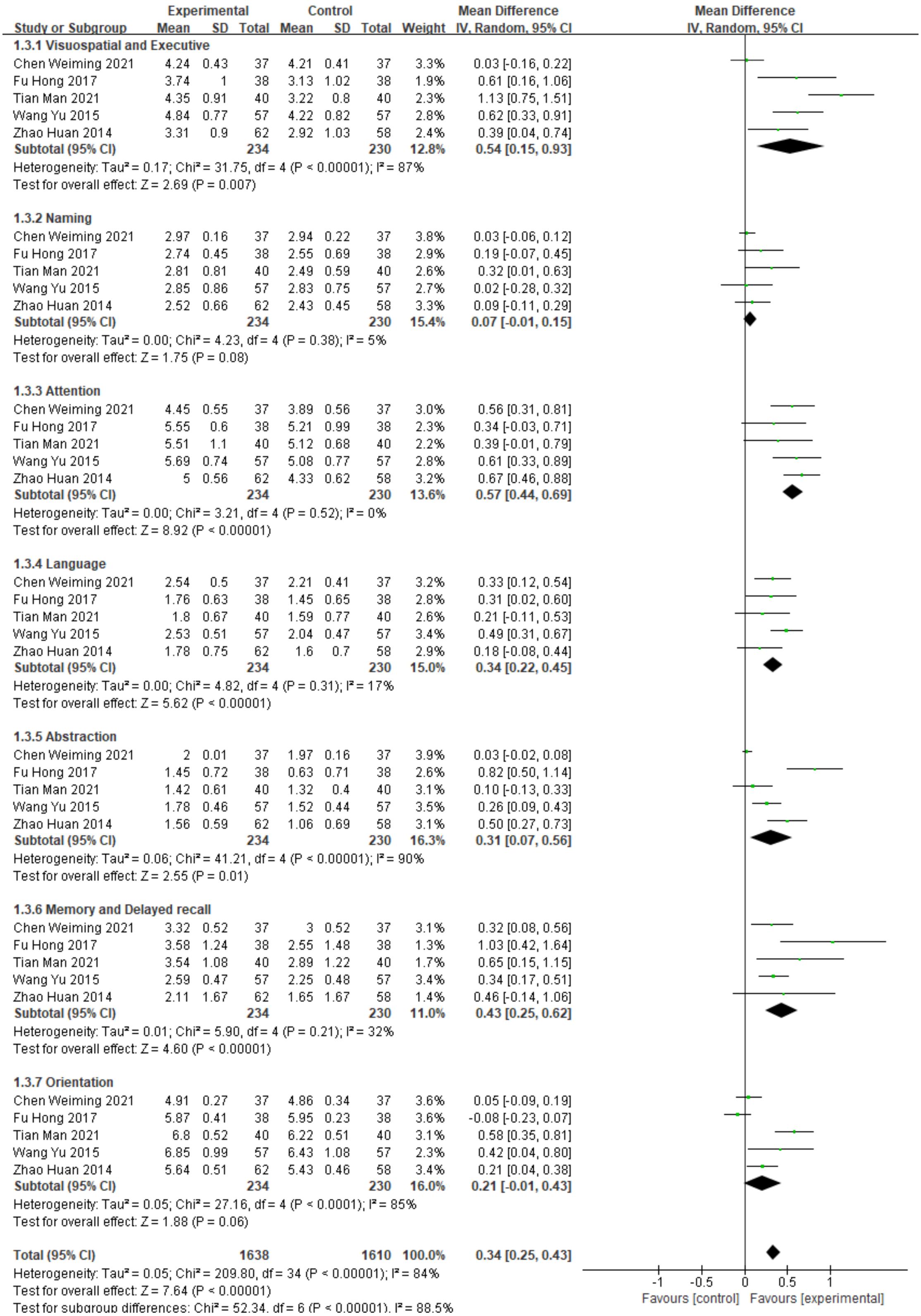

Supplement: Supplementary file 1 [file DataSheet2.PDF]
