# Supplementary material for: Efficacy and safety of the integration of traditional Chinese medicine and western medicine in the treatment of diabetes-associated cognitive decline: a systematic review and meta-analysis
Source: Front Pharmacol. 2023 Nov 22;14:1280736. doi: 10.3389/fphar.2023.1280736 (PMC10703163; doi:10.3389/fphar.2023.1280736)

Sensitivity analyses of Total Effective Rate

| Study omitted | Estimate | [95% Conf. | Interval] |
|---|---|---|---|
| Chen Weiming 2021 | 4.8319726 | 3.4546766 | 6.7583637 |
| Fu Hong 2017 | 4.6009903 | 3.2611544 | 6.4912944 |
| Gao Fengqing 2017 | 5.1319542 | 3.6270349 | 7.2612901 |
| Jin Shuoguo 2013 | 4.7780328 | 3.4217904 | 6.6718283 |
| Li Huiling 2022 | 5.4773812 | 3.8500388 | 7.792573 |
| Li Kaige 2016 | 5.3455353 | 3.762002 | 7.5956225 |
| Li Quan 2022 | 5.0130758 | 3.5776315 | 7.0244603 |
| Liu Yafen 2016 | 4.8672795 | 3.4519851 | 6.8628373 |
| Mao Ye 2019 | 5.0356464 | 3.5794909 | 7.0841737 |
| Wang Xiaoyan 2015 | 4.9677305 | 3.5549314 | 6.9420033 |
| Yang Fan 2017 | 4.9631796 | 3.5421004 | 6.9543915 |
| Yu Jinxin 2022 | 4.24053 | 3.0044594 | 5.9851341 |
| Zhang Wei 2016 | 5.0772028 | 3.6156917 | 7.1294765 |
| Combined | 4.9364129 | 3.5556001 | 6.8534628 |

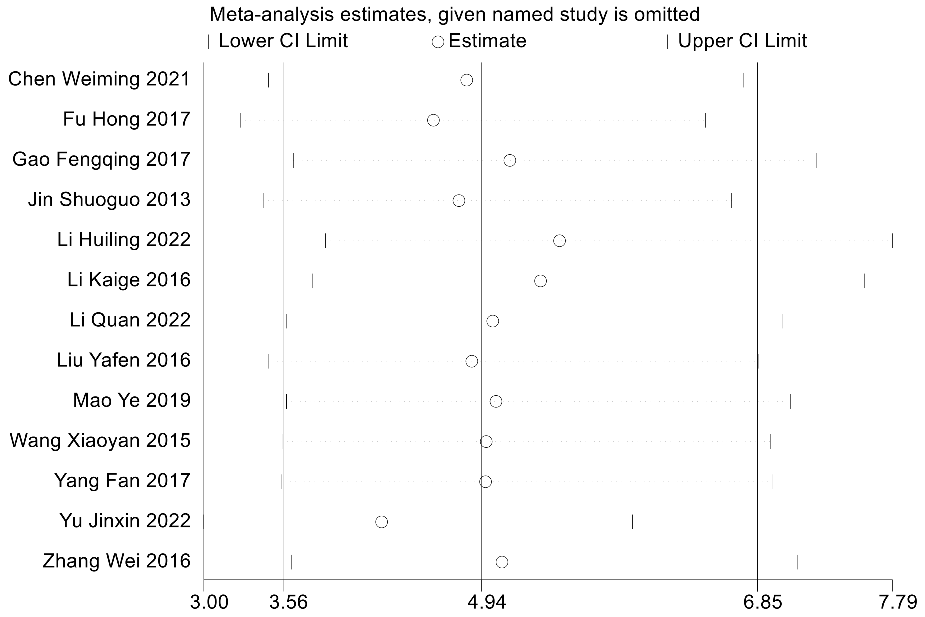

Publication bias of Total Effective Rate

```
Tests for Publication Bias

Begg's Test

  adj. Kendall's Score (P-Q) =      30
          Std. Dev. of Score =   16.39
           Number of Studies =      13
                          z  =    1.83
                    Pr > |z| =   0.067
                          z  =    1.77 (continuity corrected)
                    Pr > |z| =   0.077 (continuity corrected)

Egger's test
```

| Std_Eff | Coefficient | Std. err. | t | P>\|t\| | [95% conf. | interval] |
|---|---|---|---|---|---|---|
| slope | -.5878002 | .5112487 | -1.15 | 0.275 | -1.713051 | .5374506 |
| bias | 1.585338 | .8264209 | 1.92 | 0.081 | -.2336019 | 3.404278 |

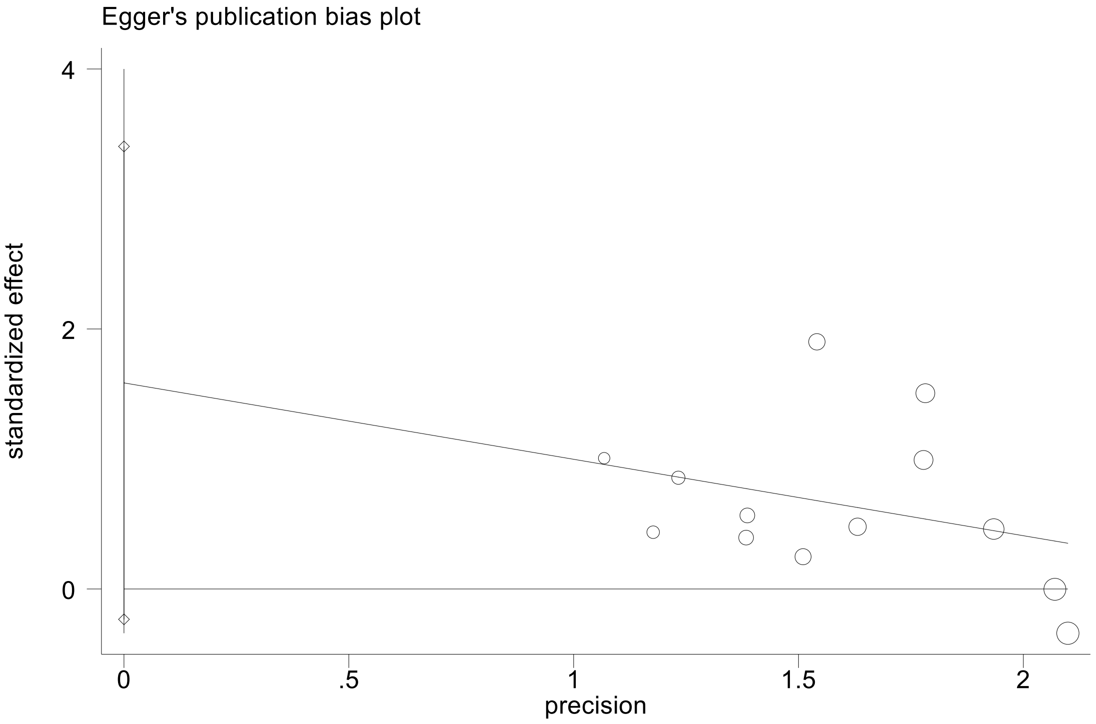

Sensitivity analyses of FPG

| Study omitted | Estimate | [95% Conf. | Interval] |
|---|---|---|---|
| Chen Weiming 2021 | -.42413166 | -.642703 | -.20556033 |
| Fu Hong 2017 | -.33931547 | -.57529634 | -.10333461 |
| Li Huiling 2022 | -.34310201 | -.5879373 | -.09826671 |
| Li Quan 2022 | -.39558437 | -.63344276 | -.15772597 |
| Wang Kaijun 2016 | -.31171432 | -.5209707 | -.10245795 |
| Wang Yu 2015 | -.39736843 | -.64079678 | -.1539401 |
| Yang Fan 2017 | -.34881812 | -.58700043 | -.11063585 |
| Yan Xiaoyan 2019 | -.34679097 | -.58404386 | -.10953803 |
| Yu Jinxin 2018 | -.3845914 | -.62374675 | -.14543602 |
| Zhang Wei 2016 | -.43060723 | -.63939095 | -.22182351 |
| Combined | -.37248444 | -.59228313 | -.15268576 |

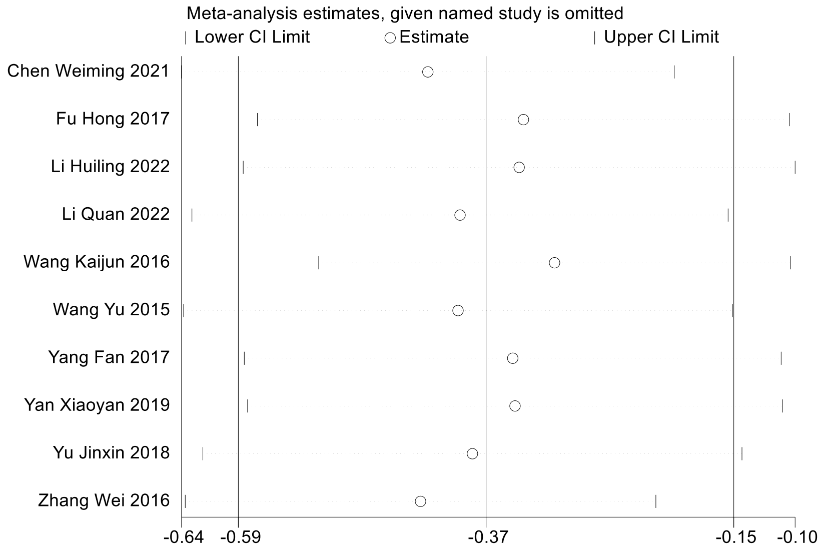

Publication bias of FPG

```
Begg's Test

  adj. Kendall's Score (P-Q) =       5
          Std. Dev. of Score =   11.18
           Number of Studies =      10
                          z  =    0.45
                    Pr > |z| =   0.655
                          z  =    0.36 (continuity corrected)
                    Pr > |z| =   0.721 (continuity corrected)
```

Egger's test

| Std_Eff | Coefficient | Std. err. | t | P>\|t\| | [95% conf. | interval] |
|---|---|---|---|---|---|---|
| slope | -.9245383 | .7666118 | -1.21 | 0.262 | -2.692348 | .8432717 |
| bias | 2.37796 | 3.351617 | 0.71 | 0.498 | -5.350883 | 10.1068 |

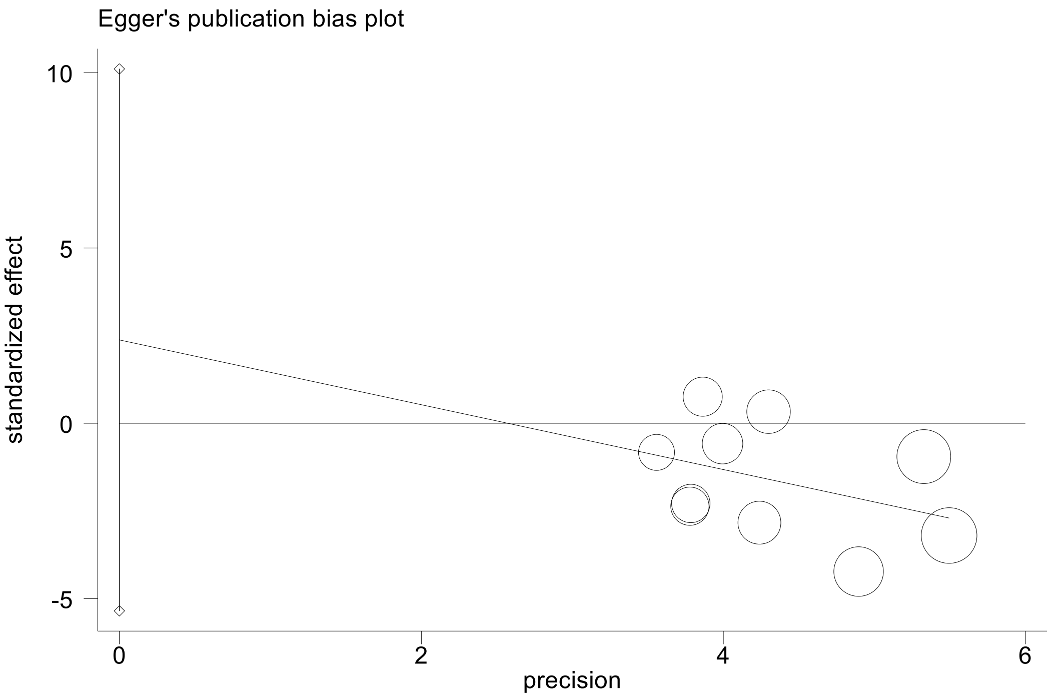

Sensitivity analyses of HbA1c

| Study omitted | Estimate | [95% Conf. | Interval] |
|---|---|---|---|
| Chen Weiming 2021 | -1.0256155 | -1.5029255 | -.54830533 |
| Fu Hong 2017 | -1.047478 | -1.5213515 | -.57360452 |
| Li Huiling 2022 | -.99572527 | -1.4834908 | -.50795972 |
| Li Quan 2022 | -1.0921906 | -1.5364518 | -.64792949 |
| Wang Kaijun 2016 | -.92883682 | -1.372458 | -.4852156 |
| Wang Yu 2015 | -1.0607816 | -1.5360672 | -.58549583 |
| Yang Fan 2017 | -.79256922 | -1.1263821 | -.45875633 |
| Yan Xiaoyan 2019 | -1.0295117 | -1.5018456 | -.55717772 |
| Yu Jinxin 2018 | -1.0285861 | -1.4978151 | -.55935711 |
| Yu Jinxin 2022 | -.93498081 | -1.384177 | -.48578471 |
| Zhang Wei 2016 | -1.0842752 | -1.5352502 | -.63330042 |
| Combined | -.99983825 | -1.4312761 | -.56840036 |

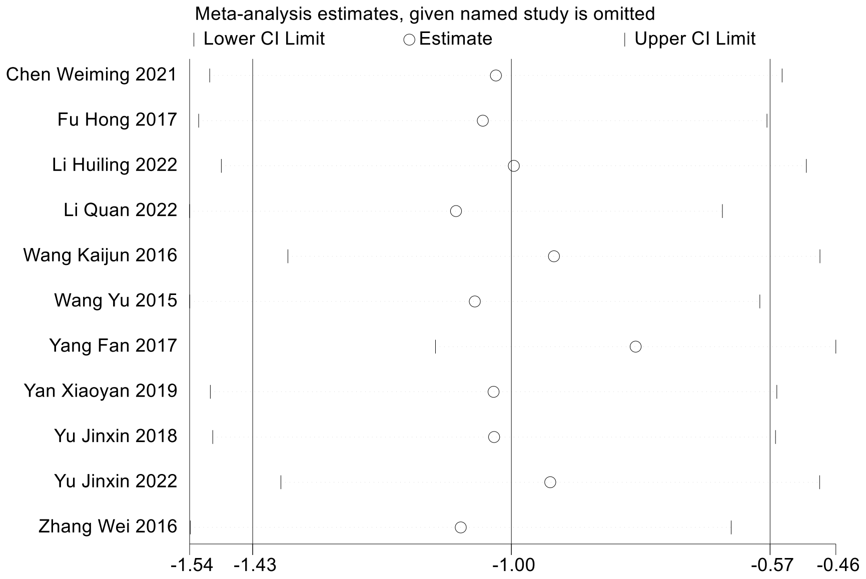

Publication bias of HbA1c

```
Begg's Test

  adj. Kendall's Score (P-Q) =      -9
          Std. Dev. of Score =   12.85
           Number of Studies =      11
                          z  =   -0.70
                    Pr > |z| =   0.484
                          z  =    0.62 (continuity corrected)
                    Pr > |z| =   0.533 (continuity corrected)
```

Egger's test

| Std_Eff | Coefficient | Std. err. | t | P>\|t\| | [95% conf. interval] | |
|---|---|---|---|---|---|---|
| slope | .7826921 | 1.107507 | 0.71 | 0.498 | -1.722664 | 3.288048 |
| bias | -7.001255 | 4.570645 | -1.53 | 0.160 | -17.34077 | 3.338262 |

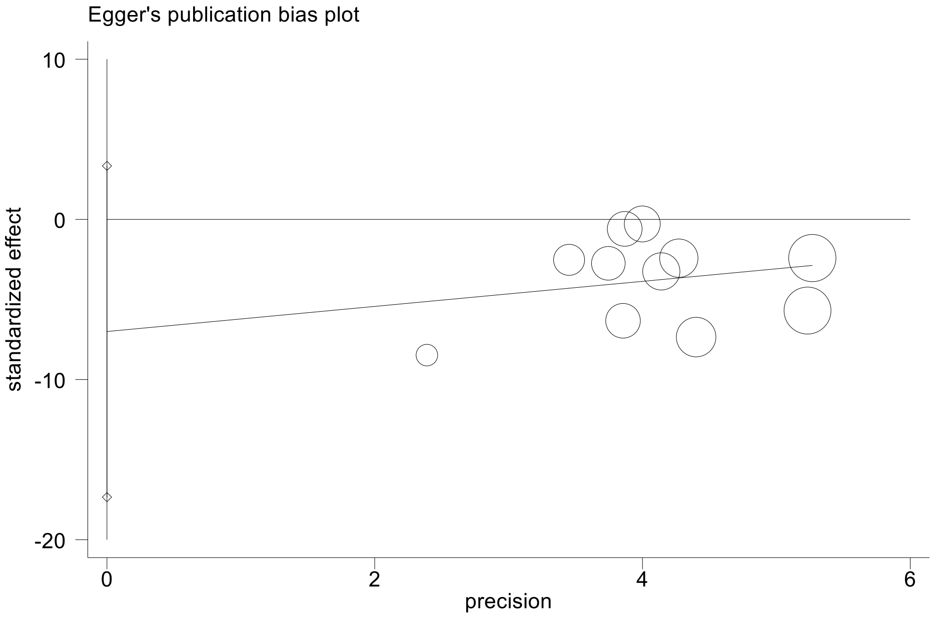

Sensitivity analyses of MoCA Score

| Study omitted | Estimate | [95% Conf. | Interval] |
|---|---|---|---|
| Chen Weiming 2021 | .89753705 | .65653712 | 1.1385369 |
| Fu Hong 2017 | .99442893 | .68975073 | 1.2991071 |
| Gao Fengqing 2017 | .91575867 | .65442204 | 1.1770953 |
| Jin Shuoguo 2013 | 1.0358206 | .75071353 | 1.3209276 |
| Li Huiling 2022 | .9560768 | .6560306 | 1.2561231 |
| Li Kaige 2016 | .99421591 | .68454039 | 1.3038913 |
| Li Quan 2022 | 1.0300514 | .73698801 | 1.3231148 |
| Tian Man 2021 | .97987145 | .67559618 | 1.2841468 |
| Wang Yu 2015 | 1.0360721 | .7448014 | 1.3273429 |
| Yang Fan 2017 | .99568009 | .69394141 | 1.2974188 |
| Yu Jinxin 2022 | 1.013711 | .71170461 | 1.3157173 |
| Zhao Huan 2014 (2) | 1.0231714 | .72151977 | 1.3248229 |
| Combined | .98907349 | .70983307 | 1.2683139 |

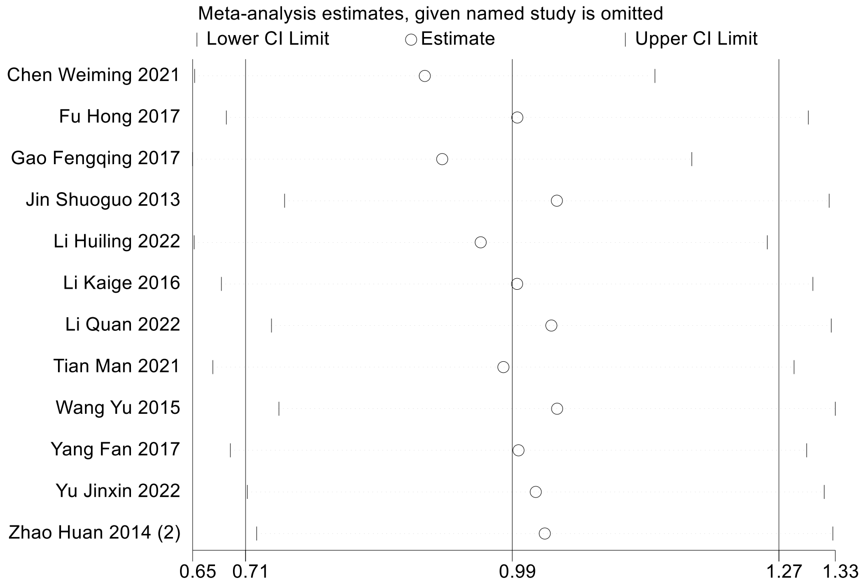

Publication bias of MoCA Score

```
Tests for Publication Bias

Begg's Test

  adj. Kendall's Score (P-Q) =       4
          Std. Dev. of Score =   14.58
           Number of Studies =      12
                          z  =    0.27
                    Pr > |z| =   0.784
                          z  =    0.21 (continuity corrected)
                    Pr > |z| =   0.837 (continuity corrected)
```

Egger's test

| Std_Eff | Coefficient | Std. err. | t | P>\|t\| | [95% conf. | interval] |
|---|---|---|---|---|---|---|
| slope | .741099 | .8400837 | 0.88 | 0.398 | -1.130724 | 2.612922 |
| bias | 1.04606 | 3.666073 | 0.29 | 0.781 | -7.12246 | 9.214581 |

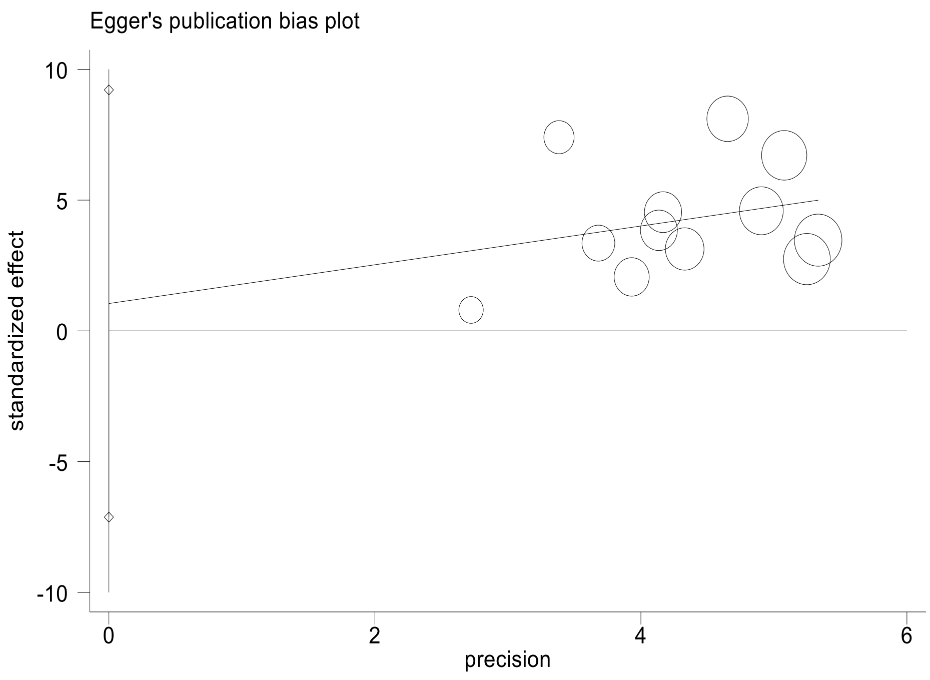

Sensitivity analyses of MMSE Score

| Study omitted | Estimate | [95% Conf. | Interval] |
|---|---|---|---|
| 蔡祥飞2014 | -1.2446291 | -1.8572363 | -.63202196 |
| 金硕果2013 | -1.3428127 | -1.9520704 | -.73355502 |
| 李全2022 | -1.3725233 | -2.0052261 | -.7398206 |
| 杨帆2017 | -1.4186319 | -2.0102761 | -.82698792 |
| 俞金鑫2022 | -1.1615818 | -1.7155188 | -.60764474 |
| 张伟2016 | -1.0677764 | -1.5152645 | -.62028831 |
| 赵欢2014 | -1.382239 | -2.0267901 | -.73768783 |
| Combined | -1.2833328 | -1.8239306 | -.74273498 |

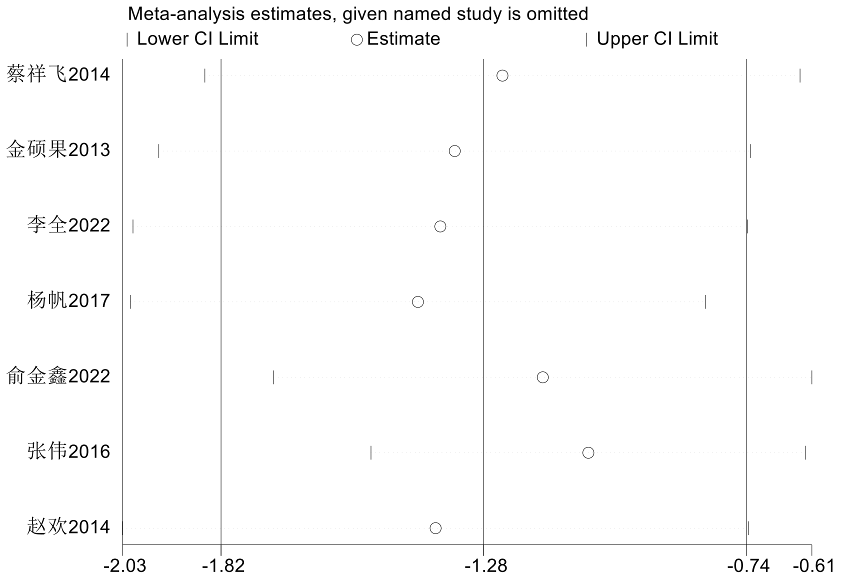

Publication bias of MMSE Score

```
Tests for Publication Bias

Begg's Test

  adj. Kendall's Score (P-Q) =      -9
          Std. Dev. of Score =    6.66
           Number of Studies =       7
                          z  =   -1.35
                    Pr > |z| =   0.176
                          z  =    1.20 (continuity corrected)
                    Pr > |z| =   0.230 (continuity corrected)

Egger's test
```

| Std_Eff | Coefficient | Std. err. | t | P>\|t\| | [95% conf. | interval] |
|---|---|---|---|---|---|---|
| slope | .3653762 | 1.085511 | 0.34 | 0.750 | -2.425018 | 3.15577 |
| bias | -5.690172 | 3.984438 | -1.43 | 0.213 | -15.9325 | 4.552151 |

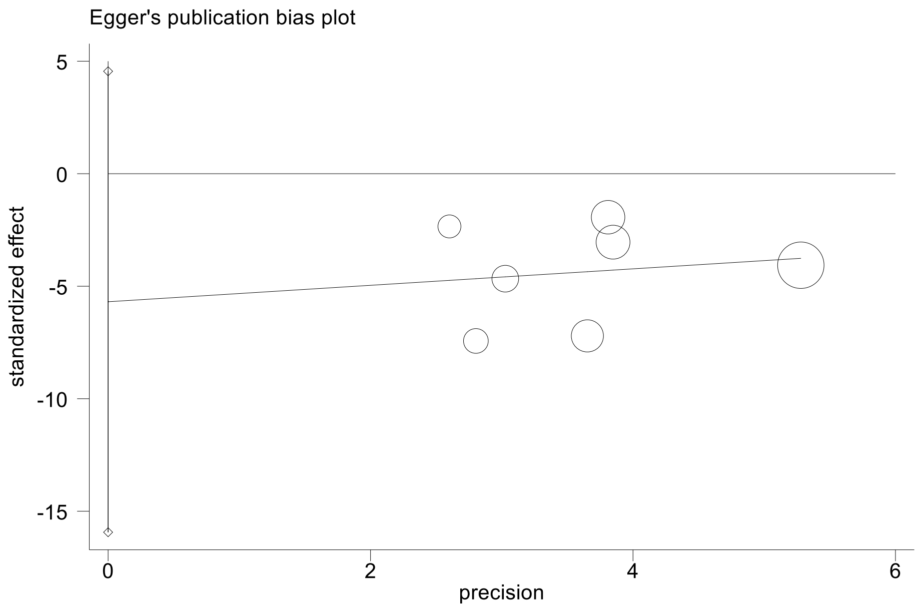

Sensitivity analyses of TNF-α

```
. metaninf Ttotal Tmean Tsd Ctotal Cmean Csd, label(namevar=Study) random cohen
```

| Study omitted | Estimate | [95% Conf. | Interval] |
|---|---|---|---|
| Li Huiling 2022 | -1.0231252 | -1.3230436 | -.72320676 |
| Mao Ye 2019 | -1.4341015 | -2.4446151 | -.42358783 |
| Wang Yu 2015 | -1.572082 | -2.3346434 | -.80952054 |
| Combined | -1.3487406 | -1.9769098 | -.72057141 |

Publication bias of TNF-α

```
Tests for Publication Bias

Begg's Test

  adj. Kendall's Score (P-Q) =      -1
          Std. Dev. of Score =    1.91
           Number of Studies =       3
                          z  =   -0.52
                    Pr > |z| =   0.602
                          z  =    0.00 (continuity corrected)
                    Pr > |z| =   1.000 (continuity corrected)
```

Egger's test

| Std_Eff | Coefficient | Std. err. | t | P>\|t\| | [95% conf. | interval] |
|---|---|---|---|---|---|---|
| slope | .1165678 | 5.156311 | 0.02 | 0.986 | -65.40058 | 65.63371 |
| bias | -6.710076 | 23.80126 | -0.28 | 0.825 | -309.1338 | 295.7136 |

Sensitivity analyses of Adverse Reactions

| Study omitted | Estimate | [95% Conf. | Interval] |
|---|---|---|---|
| Fu Hong 2017 | .73024482 | .41535643 | 1.2838552 |
| Gao Fengqing 2017 | .30664358 | .17574538 | .53503704 |
| Jin Shuoguo 2013 | .38849035 | .2331156 | .64742446 |
| Li Quan 2022 | .42970774 | .25949189 | .71157801 |
| Yu Jinxin 2022 | .48068392 | .29023138 | .79611319 |
| Zhao Huan 2014 (2) | .32212079 | .18104225 | .57313579 |
| Combined | .42335645 | .2609466 | .68684812 |

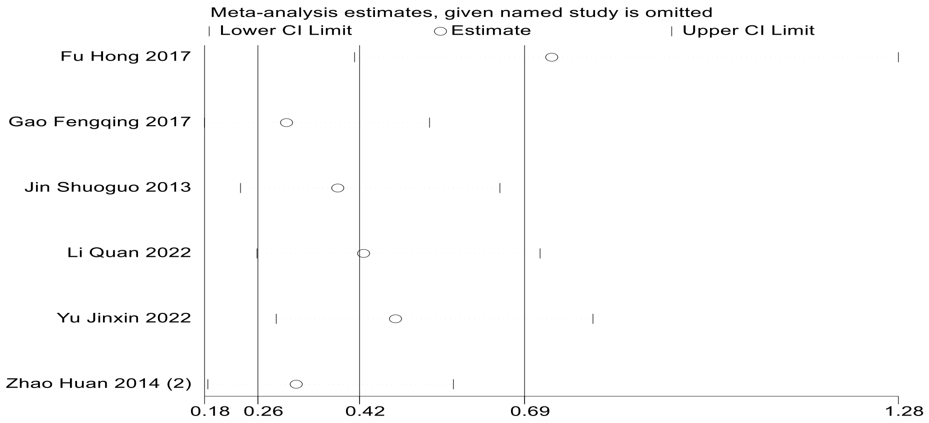

Publication bias of Adverse Reactions

```
Tests for Publication Bias

Begg's Test

  adj. Kendall's Score (P-Q) =       -7
          Std. Dev. of Score =     5.32
           Number of Studies =        6
                          z  =    -1.32
                    Pr > |z| =    0.188
                          z  =     1.13 (continuity corrected)
                    Pr > |z| =    0.260 (continuity corrected)

Egger's test
```

| Std_Eff | Coefficient | Std. err. | t | P>\|t\| | [95% conf. interval] | |
|---|---|---|---|---|---|---|
| slope | 1.53366 | 2.25322 | 0.68 | 0.533 | -4.722281 | 7.789601 |
| bias | -3.325506 | 3.268349 | -1.02 | 0.366 | -12.3999 | 5.748887 |

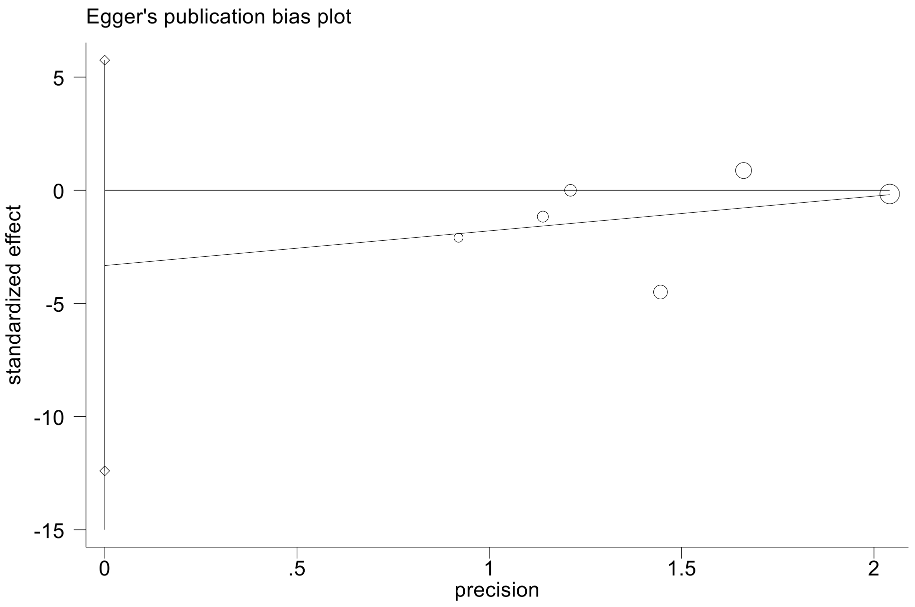

Supplement: Supplementary file 2 [file DataSheet3.PDF]
