# Supplementary material for: Efficacy and safety of the integration of traditional Chinese medicine and western medicine in the treatment of diabetes-associated cognitive decline: a systematic review and meta-analysis
Source: Front Pharmacol. 2023 Nov 22;14:1280736. doi: 10.3389/fphar.2023.1280736 (PMC10703163; doi:10.3389/fphar.2023.1280736)
Supplement: Supplementary file 3 [file DataSheet1.PDF]

Details of Search Strategy

Source: PubMed

Search	Query
#1	"Diabetes-associated Cognitive Decline" [Mesh]
#2	Diabetes-associated Cognitive Decline [Title/Abstract]
#3	Diabetic cognitive impairment [Title/Abstract]
#4	DCD [Title/Abstract]
#5	DCI [Title/Abstract]
#6	#1 OR #2 OR #3 OR #4 OR #5
#7	Chinese herbal medicine [Title/Abstract]
#8	Chinese traditional [Title/Abstract]
#9	Oriental traditional [Title/Abstract]
#10	Traditional Chinese medicine [Title/Abstract]
#11	Traditional Chinese medicinal materials [Title/Abstract]
#12	Chinese herb* [Title/Abstract]
#13	Herbal medicine [Title/Abstract]
#14	Herbal decoction [Title/Abstract]
#15	Tang [Title/Abstract]
#16	Pill [Title/Abstract]
#17	Wan [Title/Abstract]
#18	Powder [Title/Abstract]
#19	Formula [Title/Abstract]
#20	Granule [Title/Abstract]
#21	Capsule [Title/Abstract]
#22	Particles [Title/Abstract]
#23	Ointment [Title/Abstract]
#24	Prescription [Title/Abstract]
#25	Receipt [Title/Abstract]
#26	#7 OR #8 OR #9 OR #10 OR #11 OR #12 OR #13 OR #14 OR #15 OR #16 OR #17 OR #18 OR #19 OR #20 OR #21 OR #22 OR #23 OR#24 OR #25 OR #26 OR #27 OR #28 OR#29 OR#30 OR#31
#27	Random controlled trial [Title/Abstract]
#28	RCT [Title/Abstract]
#29	Random [Title/Abstract]
#30	Placebo [Title/Abstract]
#31	#33 OR #34 OR #35 OR #36
#32	#6 AND #26 AND #31

Source: Cochrane Library

Search	Query
#1	MeSH descriptor: [Diabetes-associated Cognitive Decline] explode all trees
#2	(Diabetes-associated Cognitive Decline): ti,ab,kw
#3	(Diabetic cognitive impairment): ti,ab,kw
#4	(DCD): ti,ab,kw
#5	(DCI): ti,ab,kw
#6	#1 OR #2 OR #3 OR #4 OR #5
#7	(Chinese herbal medicine): ti,ab,kw
#8	(Chinese traditional): ti,ab,kw
#9	(Oriental traditional): ti,ab,kw
#10	(Traditional Chinese medicine): ti,ab,kw
#11	(Traditional Chinese medicinal materials): ti,ab,kw
#12	(Chinese herb*): ti,ab,kw
#13	(Herbal medicine): ti,ab,kw
#14	(Herbal decoction): ti,ab,kw
#15	(Tang): ti,ab,kw
#16	(Pill): ti,ab,kw
#17	(Wan): ti,ab,kw
#18	(Powder): ti,ab,kw
#19	(Formula): ti,ab,kw
#20	(Granule): ti,ab,kw
#21	(Capsule): ti,ab,kw
#22	(Particles): ti,ab,kw
#23	(Ointment): ti,ab,kw
#24	(Prescription): ti,ab,kw
#25	(Receipt): ti,ab,kw
#26	#7 OR #8 OR #9 OR #10 OR #11 OR #12 OR #13 OR #14 OR #15 OR #16 OR #17 OR #18 OR #19 OR #20 OR #21 OR #22 OR #23 OR#24 OR #25 OR #26 OR #27 OR #28 OR#29 OR#30 OR#31
#27	(Random controlled trial): ti,ab,kw
#28	(RCT): ti,ab,kw
#29	(Random): ti,ab,kw
#30	(Placebo): ti,ab,kw
#31	#33 OR #34 OR #35 OR #36
#32	#6 AND #26 AND #31

Source: Embase

Search	Query
#1	'Diabetes-associated Cognitive Decline '/exp
#2	'Diabetes-associated Cognitive Decline': ab, ti
#3	'Diabetic cognitive impairment': ab, ti
#4	'DCD': ab, ti
#5	'DCI': ab, ti
#6	#1 OR #2 OR #3 OR #4 OR #5
#7	'Chinese herbal medicine': ab, ti
#8	'Chinese traditional': ab, ti
#9	'Oriental traditional': ab, ti
#10	'Traditional Chinese medicine': ab, ti
#11	'Traditional Chinese medicinal materials': ab, ti
#12	'Chinese herb': ab, ti
#13	'Herbal medicine': ab, ti
#14	'Herbal decoction': ab, ti
#15	'Tang': ab, ti
#16	'Pill': ab, ti
#17	'Wan': ab, ti
#18	'Powder': ab, ti
#19	'Formula': ab, ti
#20	'Granule': ab, ti
#21	'Capsule': ab, ti
#22	'Particles': ab, ti
#23	'Ointment': ab, ti
#24	'Prescription': ab, ti
#25	'Receipt': ab, ti
#26	#7 OR #8 OR #9 OR #10 OR #11 OR #12 OR #13 OR #14 OR #15 OR #16 OR #17 OR #18 OR #19 OR #20 OR #21 OR #22 OR #23 OR#24 OR #25 OR #26 OR #27 OR #28 OR#29 OR#30 OR#31
#27	'Random controlled trial): ab, ti
#28	'RCT): ab, ti
#29	'Random): ab, ti
#30	'Placebo): ab, ti
#31	#33 OR #34 OR #35 OR #36
#32	#6 AND #26 AND #31

Source: Web of Science

Search	Query
#1	TS="Diabetes-associated Cognitive Decline"
#2	TS="Diabetes-associated Cognitive Decline"
#3	TS="Diabetic cognitive impairment"
#4	TS="DCD"
#5	TS="DCI"
#6	#1 OR #2 OR #3 OR #4 OR #5
#7	TS="Chinese herbal medicine"
#8	TS="Chinese traditional"
#9	TS="Oriental traditional"
#10	TS="Traditional Chinese medicine"
#11	TS="Traditional Chinese medicinal materials"
#12	TS="Chinese herb"
#13	TS="Herbal medicine"
#14	TS="Herbal decoction"
#15	TS="Tang"
#16	TS="Pill"
#17	TS="Wan"
#18	TS="Powder"
#19	TS="Formula"
#20	TS="Granule"
#21	TS="Capsule"
#22	TS="Particles"
#23	TS="Ointment"
#24	TS="Prescription"
#25	TS="Receipt"
#26	#7 OR #8 OR #9 OR #10 OR #11 OR #12 OR #13 OR #14 OR #15 OR #16 OR #17 OR #18 OR #19 OR #20 OR #21 OR #22 OR #23 OR#24 OR #25 OR #26 OR #27 OR #28 OR#29 OR#30 OR#31
#27	TS="Random controlled trial"
#28	TS="RCT"
#29	TS="Random"
#30	TS="Placebo"
#31	#33 OR #34 OR #35 OR #36
#32	#6 AND #26 AND #31

CNKI

(SU = '糖尿病认知功能障碍' OR SU='糖尿病认知功能损害') AND (FT='中医' OR FT='中药' OR FT='中医药' OR FT='中西医' OR FT='中成药' OR FT='汤' OR FT='片' OR FT='丸' OR FT='散' OR FT='胶囊' OR FT='颗粒' OR FT='水' OR FT='液' OR FT='合剂' OR FT='注射液') AND (FT='随机')

VIP

(M=糖尿病认知功能障碍 OR 糖尿病认知功能损害) AND ((U=中医 OR 中药 OR 中西医 OR 中成药 OR 汤 OR 片 OR 丸 OR 散 OR 胶囊 OR 颗粒 OR 水 OR 液 OR 合剂 OR 注射液) OR (R=中医 OR 中药 OR 中西医 OR 中成药 OR 汤 OR 片 OR 丸 OR 散 OR 胶囊 OR 颗粒 OR 水 OR 液 OR 合剂 OR 注射液)) AND ((U=随机) OR (R=随机))

Wanfang

全部:(("糖尿病认知功能障碍" OR "糖尿病认知功能损害") AND ("中医" OR "中药" OR "中西医" OR "中成药" OR "汤" OR "片" OR "丸" OR "散" OR "胶囊" OR "颗粒" OR "水" OR "液" OR "合剂" OR "注射液") AND ("随机")

CBM

(("糖尿病认知功能障碍"[全部字段:智能] OR "糖尿病认知功能损害"[全部字段:智能])) AND(("中医"[全部字段:智能] OR "中药"[全部字段:智能] OR "中西医"[全部字段: 智能] OR ("中成药"[常用字段:智能] OR "汤"[全部字段:智能]) OR "片"[全部字段:智能] OR "丸"[全部字段:智能] OR "散"[全部字段:智能] OR "胶囊"[全部字段:智能] OR "颗粒"[全部字段:智能] OR "水"[全部字段:智能] OR"液"[全部字段:智能] OR "合剂"[全部字段:智能] OR "注射液"[全部字段:智能])) AND ("随机"[全部字段:智能])
